# Comparing machine learning approaches for estimating soil saturated hydraulic conductivity

**DOI:** 10.1371/journal.pone.0310622

**Published:** 2024-11-14

**Authors:** Ali Akbar Moosavi, Mohammad Amin Nematollahi, Mohammad Omidifard

**Affiliations:** 1 Faculty of Agriculture, Department of Soil Science and Engineering, Shiraz University, Shiraz, IR Iran; 2 Faculty of Agriculture, Department of Biosystems Engineering, Shiraz University, Shiraz, IR Iran; Euro-Mediterranean Center for Climate Change: Fondazione Centro Euro-Mediterraneo sui Cambiamenti Climatici, ITALY

## Abstract

Characterization of near (field) saturated hydraulic conductivity (K_fs_) of the soil environment is among the crucial components of hydrological modeling frameworks. Since the associated laboratory/field experiments are time-consuming and labor-intensive, pedotransfer functions (PTFs) that rely on statistical predictors are usually integrated with the existing measurements to predict K_fs_ in other areas of the field. In this study some of the most appropriate machine learning approaches, including variants of artificial neural networks (ANNs) were used for predicting K_fs_ by some easily measurable soil attributes. The analyses were performed using 100 measurements in Bajgah Agricultural Experimental Station. First, physico-chemical inputs as bulk density (BD), initial water content (W_i_), saturated water content (W_s_), mean weight diameter (MWD), and geometric mean diameter (GMD) of aggregates, pH, electrical conductivity (EC), and calcium carbonate equivalent (CCE) were measured. Then, radial basis functions (RBFNNs), multilayer perceptron (MLPNNs), hybrid genetic algorithm (GA-NNs), and particle swarm optimization (PSO-NNs) neural networks were utilized to develop PTFs and compared their accuracy with the traditional regression model (MLR) using statistical indices. The statistical assessment indicated that PSO-NNs with the lowest RMSE and MAPE as well as the highest correlation coefficient (R) value provided the most accurate and robust prediction of K_fs_. The prediction models ranked as PSO-NNs (R = 0.958; RMSE = 0.343; MAPE = 9.47), GA-NNs (R = 0.949; RMSE = 0.404; MAPE = 11.83), MLPNNs (R = 0.933; RMSE = 0.426; MAPE = 12.13), RBFNNs (R = 0.926; RMSE = 0.452; MAPE = 14.30), and MLR (R = 0.675; RMSE = 0.685; MAPE = 22.54) in terms of their performances for the test data set. Results revealed that all NN models particularly PSO-NNs were efficient in prediction of K_fs_. However, further evaluations may be recommended for other soil conditions and input variables to quantify their potential uncertainties and wider potential and versatility before they are used in other geographical locations/soil conditions.

## Introduction

Recently, the water flow process and water distribution in the soil environment have received increased attention from soil scientists, hydrologists, and hydrogeologists worldwide due to increased environmental awareness [1]. Hydraulic attributes (e.g. unsaturated and saturated hydraulic conductivity) play key roles in the mentioned processes. In other words, the hydraulic properties of soils are the most significant parameters in hydrological processes like groundwater recharge, irrigation or drainage, soil and water conservation practices, solutes and contaminants transportation within the soil profile, and reclamation of saline-sodic soils. Therefore, hydraulic soil properties are vital parameters in the soil models in which the mentioned processes are studied. Field-saturated hydraulic conductivity, *K*_*fs*_ is one of the fundamental soil hydraulic attributes characterizing water flow through soil. Besides, it is an important soil parameter controlling steady ponded infiltration [2]. It is also used to derive several other soil parameters (e.g., the alpha parameter, mean pore radius, sorptivity coefficient, the wetting-front potential, and the macroscopic capillary length) [2]. Consequently, an accurate investigation of the mentioned hydraulic attribute is of vital significance [3].

On one hand, the laboratory or field determination of the soil hydraulic properties is usually expensive and painstaking [4]. On the other hand, due to their high inherent spatiotemporal variability, a large number of samples is needed for their reliable and proper determination [3,5]. To put it another way, because of the mentioned problems associated with the field and laboratory tests, therefore there is a need for other determining methods. Most investigators prefer to predict the hydraulic attributes using pedotransfer functions (PTFs) which are the mathematical relationships between the easily available soil parameters (e.g., bulk density, organic matter, and textural components) and hydraulic soil attributes [4,6,7]. Several PTFs such as those proposed by Saxton et al. [4], Zhuang et al. [8], Moosavi and Sepaskhah [9] and references cited therein have been developed previously. Artificial neural networks (ANNs) without requiring any priori assumption are the most widely used PTFs [10,11] and are widely used tools to model the complicated dependencies (input-output) and for predicting soil hydraulic attributes [7,12–14], soil moisture [15,16], and infiltration rate [17]. The idea of ANNs as a branch of intelligent systems is inspired by biological neural networks of the human brain [18] and received increased attention over the past decades. The approach has been effectively used as a powerful tool for modeling problems in different branches of science and engineering. It has been reported that ANNs have often better performance than the classical and traditional regression techniques [10]. The feed-forward NNs (FFNNs) such as radial basis function NNs (RBFNNs) and multilayer perceptron NNs (MLPNNs) are widely used as efficient tools for function approximation or pattern recognition tasks [19].

RBFNNs are the well-known and very fast learning types of ANNs that have been widely used for function approximation and classification tasks. They generally are designed quickly (i.e., in a small portion of the training time for the standard FFNNs) and in the conditions that many training samples are available, they work best [20]. Both RBFNNs and MLPNNs are strong classifiers with the ability for generalization of the vague input data. However, RBFNNs utilize a type of learning strategy that is faster than MLPNNs [21]. Since the learning algorithms (e.g., Levenberg-Marquardt and Conjugate Gradient) used in the traditional NNs maybe get stuck in the local minimums; therefore, the hybrids of Genetic Algorithm (GA) and Particle Swarm Optimization (PSO) techniques with NNs are utilized to find the best weights and biases. The GA and PSO tools are most common search-based methods, which classified as modern heuristic optimization techniques [22–24]. These methods are useful to optimize complex multivariable cases in various areas. The conjunction of GA and PSO with NNs are also called hybrid methods, which are more effective and a branch of soft computing [25–27].

The GA, PSO, and NNs have been used separately for some applications in soil and water research. For instance, GA has been applied in some previous studies for the estimation of pore geometry [28], soil electrical conductivity [29], dynamics of soil moisture [30], soil–water characteristic curve [31,32], and also for optimizing fertilizer addition [33], long-term groundwater monitoring [34], and urban drainage model parameter [35], rainfall-runoff modeling [36], and groundwater flow coupled to contaminant transport [37].

The PSO has been also used for the prediction of soil–structure interaction [38], analyzing the stability of slopes [39], calibration of parameters in soil models [40], wastewater treatment network planning [41], and identification of parameters for elastoplastic modeling of unsaturated soils [42].

There are also studies on the application of hybrid algorithms that enjoy a combination of unique advantages of PSO or GA with NNs [43]. For example, hybrid NNs with GA were used to investigate the mechanical processes of unsaturated soils [25], and to model the groundwater inflow to open pit mine [44]. Furthermore, the PSO-NNs combination was used to predict soil compaction [26] and PSO and GA with a combination of multiple regression approaches have been applied to predict the mechanical resistance of the soils [45].

Specifically, several previous studies have been performed for prediction of hydraulic characteristics of the soils (e.g., hydraulic conductivity) by NNs [3,14,46], GA [47–52], and PSO [53].

Soil hydraulic properties including saturated and unsaturated hydraulic conductivity play key roles in soil and hydrological processes. Consequently, their accurate investigation is very important [3]. Direct field and laboratory measurements are cost, laborious, and time-consuming. Therefore, their estimations have received increasing attention.

However, few studies conducted to predict these soil properties by a combination of GA and PSO with NNs [54–56]. Until now, the mentioned hybrid techniques particularly GA-NNs and PSO-NNs as novel tools have been not used for prediction of field-saturated hydraulic conductivity (K_fs_). Therefore, this study aimed to i) predict the K_fs_ of calcareous soils using RBFNNs, MLPNNs, and the hybrid techniques of GA-NNs and PSO-NNs and ii) evaluate the results of NNs models against that of the traditional multiple linear regression (MLR) approach.

## Materials and methods

### The study area and basic soil physico-chemical properties

**Fig 1:**
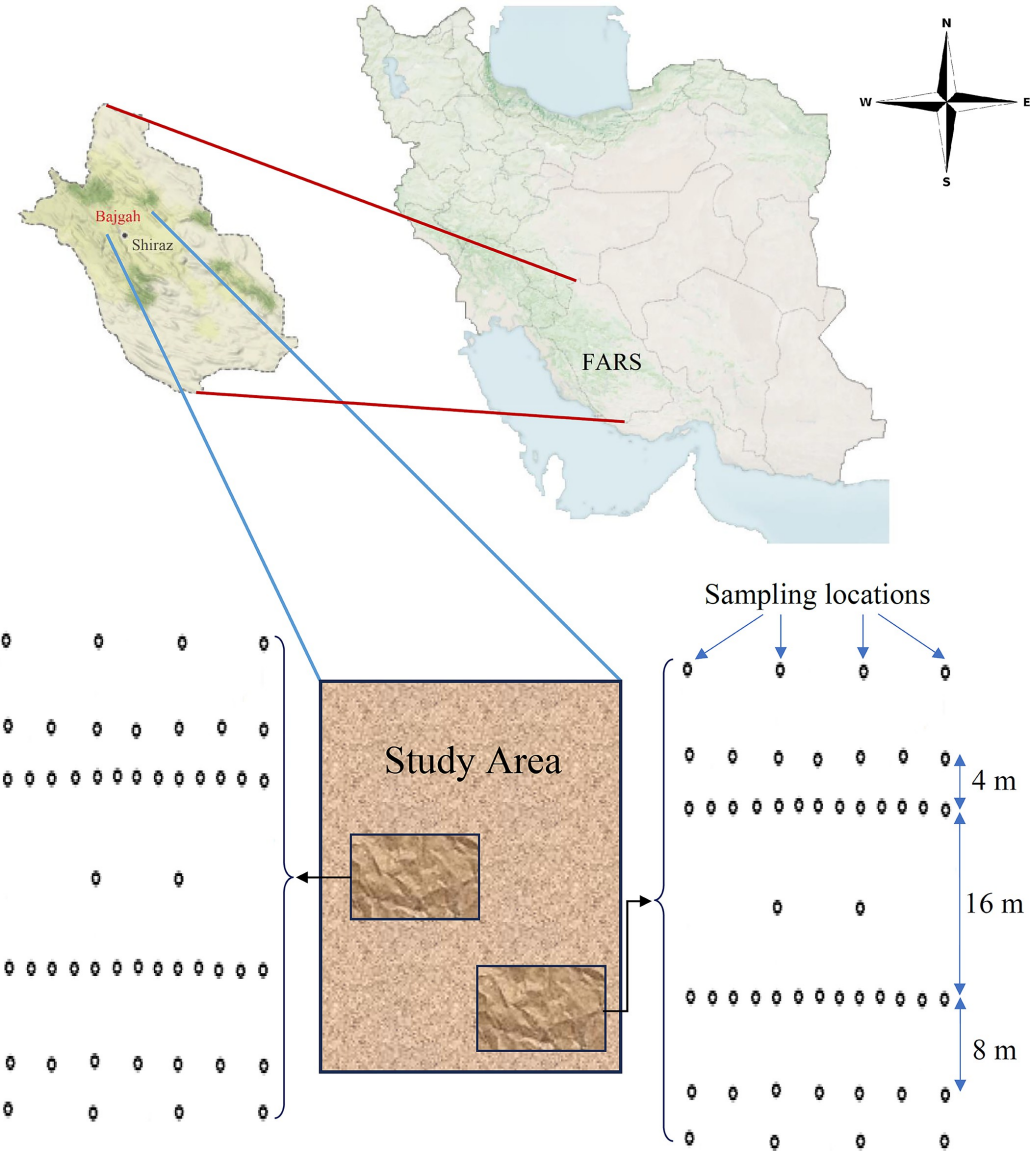
Schematic description of the study area along with the experimental locations (https://earthobservatory.nasa.gov/map#5/30.145/54.185).

This study was conducted on calcareous soils of Bajgah region, Faculty of Agriculture (Fig 1), Shiraz University, IR Iran (29˚ 44′ N, 52˚ 34′ E, 1810 m elevation above sea level). The climate, soil moisture and soil temperature regimes of the study area with a long-term mean annual temperature of 13.4 ^˚^C and precipitation of 388 mm, are classified as Thermo Mediterranean, Xeric, and Mesic, respectively [57]. The study area consisted of two calcareous soil series namely Daneshkadeh and Kooye Asatid. Table 1 shows the detailed descriptions of the soil series. According to Abtahi et al. [57], geological formation of the study area consisted of Sachun and Asmari-Jahrom formations and Kuarters depositions. In the study area, silty and gypsiferous stones, and fine marl are located in the Asmari-Jahrom formation [58].

**Table 1:** Site description and the mean value of selected soil properties.

Soil series	Bamoo	Shekarbani	Kooye-Asatid	Ramjerdi	Daneshkadeh	Pomp-Nemazi
Area (ha)	116	156	1762	296	745	568
Proportion of the study area (%)	3.16	4.25	48.04	8.07	20.31	15.49
Parent materials	Alluvial material	Alluvial-colluvial material	Gravelly calcareous alluvial and colluvial materials	Fine-grain calcareous alluvial materials	Fine-grain calcareous alluvial materials	Fine-grain calcareous alluvial materials
Soil Taxonomy classification^a^	Loamy-skeletal over fragmental, carbonatic, mesic, Typic Xerorthents	Fragmental, mixed, mesic, Typic Xerorthents	Loamy-skeletal over fragmental, carbonatic, mesic, Fluventic Xerorthents	Fine, mixed, mesic, Fluventic Xerochrepts	Fine, mixed, mesic, Calcixerollic Xerochrepts	Fine, mixed, mesic, Fluventic Xerochrepts
Slope degree (%)	3–5	3–5	3–5	1–3	<1	<1
Vegetation cover	*Reseda lutea*, *Chenopodium album*, *Vaccaria pyramidata*, *Lactuca* sp., *Euphorbia* sp., *Carthamus* sp., *Convolvulus* sp., *Turgenia* sp.	*Glycyrrhiza glabra*, *Centaurea* sp., *Lactuca* sp., *Carthamus* sp.,	*Alhagi camelorum*, *Glycyrrhiza glabra*, *Peganum harmald*, *Amygdalus sp*., *Astragalus sp*., *Carthamus sp*., *Gundelia sp*., *Lactuca sp*.	*Triticum* sp., *Convolvulus* sp., *Carthamus* sp.,	*Brassica napus*, *Hordeum vulgare*, *Zea mayze Triticum* sp.,	*Glycyrrhiza glabra*, *Alhagi camelorum Triticum* sp.,
Land use	Forest	Forest	Rangeland, Dryland farming	Dryland farming	Irrigated agriculture	Irrigated agriculture
Soil depth (cm)	100	70	110	140	>180	155
Soil color	Brownish yellow (10YR 6/4)	Yellowish brown (10YR 5/4)	Yellowish Brown (10YR 5/4)	Yellowish Brown (10YR 6/4)	Brown (10YR 5/4)	Yellowish brown (10YR 5/6)
Gravel (%)	15–75	80	35–75	-	-	-
Soil structure (stability)	Platy to Granular (very friable)	Granular (friable)	Platy to Granular (very friable)	Clody (friable)	Clody (friable)	Clody (firm)
Sand (%)	46	57	40	24	35	21
Silt (%)	41	35	47	47	35	47
Clay (%)	13	8	13	29	30	32
Soil textural class	Gravelly loam	Gravelly sandy loam	Gravelly loam	Clay loam	Clay loam	Clay loam
Saturated water content, W_s_ (%wt)	45	39	43	44	52	44
Organic matter, OM (%)	0.2	1.2	3.06	0.7	1.49	1.2
pH of saturated past	8	8.2	7.45	8	7.60	8
Electrical conductivity, EC (dS m^-1^)	0.39	0.3	0.91	0.48	0.60	0.48
Calcium-carbonate equivalent, CCE (%)	40	38	41	33	45	27
Gypsum (%)	0.02	0.03	0.02	0.01	0.01	0.02
Cation-exchange capacity (cmol_+_ kg^-1^)	51	58	56	44	48	50

^a^Soil Survey Staff [59]. Part of the data was adapted from Abtahi et al. [57] and Omidifard [60].

Infiltration experiments were conducted at 100 experimental locations. Samples were collected directly at or near each experimental location just before or after the infiltration tests to measure the physico-chemical properties of the soils. To evaluate the real influence of physico-chemical attributes on K_fs_ of the soil, disturbed soil samples from a 0 to 20 cm depth were collected immediately before infiltration tests near each experimental location for determining the initial gravimetric water (W_i_) content. To measure the saturated water content (W_s_), intact samples of 5.4 cm diameter and 3 cm height were taken directly at the experimental points immediately after infiltration tests using a core sampler. Before taking samples to the laboratory, they were kept in plastic bags to avoid water loss via evaporation [3]. The W_i_ and W_s_ of the soils were measured using the oven-drying approach and bulk density (BD) of the samples were determined using the core method. To measure the volumetric initial (θ_i_) and saturated (θ_s_) moisture contents, the W_i_ and W_s_ were multiplied by their corresponding BD values, respectively.

Besides the infiltration tests, a 3-kg soil sample was taken from 0 to 20 cm depth at experimental locations. The samples were transferred to the laboratory to determine physico-chemical soil attributes. The samples were air-dried and sieved with a 6.3 mm sieve. The size distribution of aggregates was measured using the dry-sieving approach similar to those used by other investigators [61]. The mean weight diameter (MWD) and geometric mean diameter (GMD) of aggregates were calculated using Eqs (1) and (2), respectively:

$$MWD=\sum_{i=1}^{m}f_{i}.{\bar{d}}_{i}$$ (1)

$$GMD=exp(\frac{\sum_{i=1}^{m}f_{i}.ln({\bar{d}}_{i})}{\sum_{i=1}^{m}f_{i}})$$ (2)

where ${\bar{d}}_{i}$ is the average diameter of aggregates in each range of size and f_i_ is the gravimetric proportion of aggregates in each studied range.

Part of the soil samples was sieved with a 2-mm sieve and some of the initial physico-chemical attributes of the soils were measured using the following procedures similar to the methods used by the other investigators [62–64]: Soil texture including percentage of clay, silt, and sand using hydrometer method; organic matter percentage (OM) by wet oxidation procedure; calcium-carbonate equivalent (CCE) by neutralization method with hydrochloric acid and titration with sodium hydroxide; pH by pH-meter in saturated paste; electrical conductivity (EC) by EC-meter in soil saturated extract; the concentration of soluble Mg^+2^ and Ca^+2^ by titration, and soluble Na^+^ using a flame photometer. The sodium adsorption ratio (SAR) was calculated using the following equation proposed by the U.S. Department of Agriculture Salinity Laboratory [65]:

$$\mathrm{SAR}=\frac{Na^{+1}}{\sqrt{\frac{Ca^{+2}+Mg^{+2}}{2}}}$$ (3)

where Na^+1^, Ca^+2^, and Mg^+2^ are the concentrations of their soluble forms in meq L^−1^.

### Determining field saturated hydraulic conductivity

**Fig 2:**
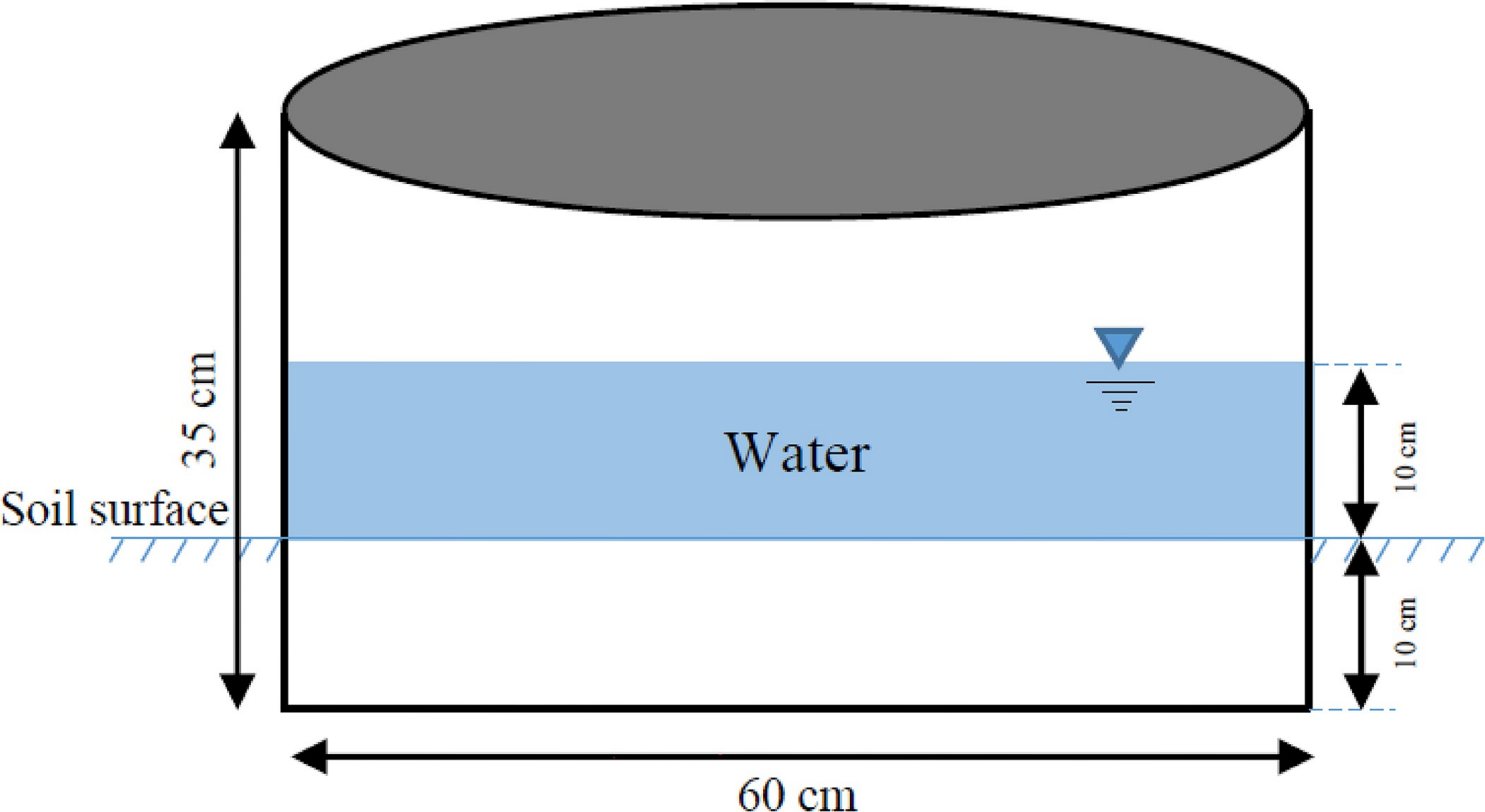
Schematic description of single ring apparatus used in this study.

In order to measure K_fs_, infiltration experiments were performed using a single-ring method [2]. At experimental locations, stones and grasses were removed from the soil surface without altering the soil structure. After that, single-ring infiltration tests were conducted using a ring of 60 cm diameter and 35 cm height (Abzar Tooseaeh Sahand Co., Tabriz, IR Iran). The ring was driven into the soil by nearly 10 cm using the hand hammer and block of wood. Then, a height of 5 cm water was supplied in the ring at falling head conditions in which the water was supplied in the ring and allowed to drop with time (Fig 2). Infiltrated water into the soil profile was measured at intervals of 3 min after supplying the water until the steady-state conditions were met. Infiltration experiment based on the abovementioned procedures was also carried out for 5, 10, 15, and 20 cm height of water (*H*). The *K*_*fs*_ was calculated using the infiltration data using the following Multiple-Ponding-Depth approach [2]:

The approach calculates *K*_*fs*_ using the following equation by using two or more *H* levels (four *H* levels in the present study):

$$K_{fs}=\frac{\gamma }{a}(\frac{dQ_{s}}{dH})$$ (4)

where $\frac{dQ_{s}}{dH}$ is the slope of the linear regression of *Q*_*s*_ *vs*. *H*. The *H* values were applied continuously and in ascending order (i.e., *H*_*4*_> *H*_*3*_> *H*_*2*_> *H*_*1*_*)* to obtain the corresponding *Q*_*s*_ values. *γ* is a "shape factor" which determined from the Richards equation and realistic flow geometry. As Reynolds and Elrick [2] stated, the *G*_*e*_ values were obtained considering the complex interactions of deepness of ring insertion (*d*), ponding depth of water in the ring (*H*), ring radius (*a*), soil capillarity, and gravity. In the mentioned approach, it was assumed that *G*_*e*_ factor is independent of the depth of ponding, *H* [2]. They proposed Eq (5) for determining *γ* (*G*_*e*_) coefficient:

$$G_{e}=\gamma =0.316(\frac{d}{a})+0.184$$ (5)

### Statistical analysis

The summary statistics including the minimum, mean value, the maximum, and coefficient-of-variation (CV) of the studied soil properties were calculated (Table 2). Since normal distribution of the data is necessary for statistical analysis and model development, for further analysis the Kolmogrov-Smirnov test was applied to check the normality of distribution for the soil properties. In the cases, which the normality assumption did not satisfy, the common transformations (e.g., natural logarithm and square root) were used. In other words, the normal (normalized) attributes were used to develop MLR, RBFNNs, MLPNNs, GA-NNs, and PSO-NNs models.

**Table 2:** Summary statistics of the measured soil chemical and physical characteristics used as input variables along with that of output (target) variable.

Data set	Statistics ^a^	Inputs ^b^															Output
		Physical parameters										Chemical parameters					
		BD	W_i_	W_s_	MWD	GMD	Silt	Fine silt	Clay	Sand		pH	EC	CCE	SAR	OM	K_fs_
**Entire** **(N = 100)**	**Min.**	1.15	0.55	18.72	0.50	0.52	43.84	2.0	11.88	14.84		7.31	0.13	34.38	0.02	1.09	1.16
	**Max.**	1.89	1.69	46.81	1.04	0.85	59.60	11.0	29.88	31.56		7.81	1.19	48.50	0.43	4.42	6.16
	**Average**	1.67	1.13	31.20	0.77	0.64	52.03	7.03	24.41	23.25		7.53	0.72	43.07	0.18	2.19	2.87
	**C.V.**	0.08	0.26	0.21	0.16	0.09	0.076	0.27	0.16	0.19		0.02	0.33	0.08	0.61	0.45	0.37
	**Mean C.V.**	**0.17**										**0.29**					
**Training** **(N = 75)**	**Min.**	1.16	0.55	18.72	0.51	0.53	43.84	2.0	11.88	14.84		7.31	0.13	35.88	0.02	1.12	1.16
	**Max.**	1.89	1.69	44.02	1.04	0.85	59.40	11.0	29.88	31.56		7.77	1.19	48.50	0.42	4.42	6.16
	**Average**	1.67	1.12	30.67	0.79	0.65	52.26	7.12	24.49	23.25		7.53	0.69	43.37	0.18	2.20	2.85
	**C.V.**	0.08	0.26	0.21	0.16	0.10	0.073	0.26	0.16	0.18		0.01	0.37	0.08	0.62	0.47	0.36
	**Mean C.V.**	**0.165**										**0.310**					
**Testing** **(N = 25)**	**Min.**	1.44	0.71	22.38	0.54	0.54	45.84	3.0	19.16	16.12		7.34	0.52	34.38	0.04	1.09	1.25
	**Max.**	1.81	1.59	46.81	0.83	0.66	59.60	8.88	28.88	30.84		7.81	0.99	47.50	0.43	3.74	5.01
	**Average**	1.65	1.18	32.92	0.72	0.62	51.31	6.75	24.17	24.52		7.51	0.79	42.14	0.19	2.16	2.92
	**C.V.**	0.07	0.28	0.21	0.15	0.06	0.086	0.31	0.13	0.24		0.02	0.20	0.09	0.61	0.43	0.41
	**Mean C.V.**	**0.17**										**0.27**					

^a^ Min. and, Max. are the minimum and the maximum values, respectively. C.V. is the coefficient of variation.

^b^ BD, W_i_, W_s_, MWD, GMD, pH, EC, CCE, SAR, OM, and *K*_*fs*_ is bulk density (g cm^-3^), initial water content (g g^-1^), saturated water content (g g^-1^), mean weight diameter (mm), geometric mean diameter (mm), pH of saturated paste, electrical conductivity (dS m^-1^), calcium carbonate equivalent (%), sodium adsorption ratio ((meq L^−1^)^0.5^), organic matter content (%), and field saturated hydraulic conductivity (cm h^-1^), respectively.

Part of the data was adapted from Omidifard [60].

### Multiple Regression Analysis (MLR)

Eq (6) shows a typical regression equation:

$$V=a+\sum_{i=1}^{N}b_{i}X_{i}$$ (6)

where *V* is the response variable (*K*_*fs*_); *b*_*1*_ to *b*_*N*_ are coefficients; *a* is intercept; *X*_*1*_ to *X*_*N*_ are inputs (basic input soil attributes); and N is the number of inputs.

The data consisted of 100 laboratory-measured EC, CCE, OM, pH, SAR, soil textural components (clay, silt, fine silt, and sand content), and W_i_. The data also consisted of 100 field-measured BD, and *K*_*fs*_ and 100 calculated MWD and GMD of soil aggregates.

At first, the entire data was divided into two subgroups. The first subgroup which consisted of 75% of the data (75 data) used to develop MLR models, and the second subgroup which consisted of 25% of data (25 data) used to test (validate) the developed models. In generating MLR model, *K*_*fs*_ (response) was considered as the dependent variable (output) and the other physico-chemical properties as independent variables (inputs) in stepwise regression procedure using MATLAB software packages (Mathworks, Natick, Massachusetts, USA, 2015). In the present study, the independent data (inputs) consisted of the fractions of soil primary particles (% clay, fine silt, and silt contents), BD, MWD, GMD, W_i_, W_s_, OM, EC, pH, CCE, and SAR.

A backward stepwise procedure was applied to derive MLR models for the training data set. In this procedure, the model parameters were adjusted so that an independent soil parameter automatically remained in the regression model by software if its influence was statistically significant (*P* < 0.05). Otherwise, the independent soil parameter was eliminated from the model. Consequently, *K*_*fs*_ was presented as a statistically significant function of previously mentioned independent (input) soil attributes.

For consistent comparisons, the same data sets were used to develop the other models (i.e., RBFNNs, MLPNNs, GA-NNs, and PSO-NNs). Furthermore, in each case, the models were validated using a test dataset not included in the training procedure.

### Artificial neural networks (ANNs) approaches

**Fig 3:**
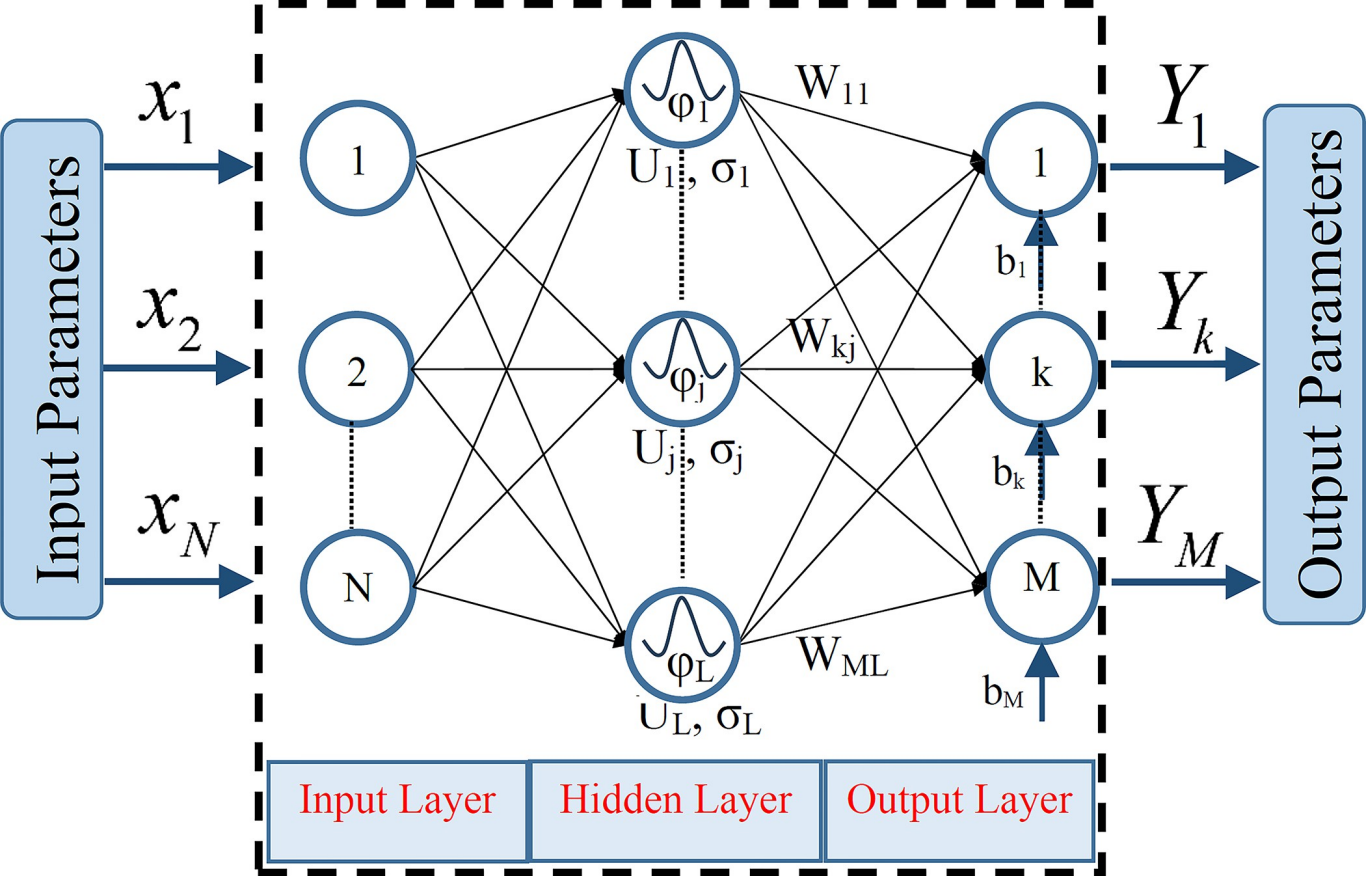
Schematic structure of the radial basis function neural networks (RBFNNs).

*Radial basis function neural networks (RBFNNs)*. RBFNNs are one of the well-known types of FFNNs for function approximation and classification applications. Its structure is composed of a two-layer that is very fast in learning (Fig 3).

The training procedure of RBFNNs consisted of the following stages: i) determining cluster centers of the input data via an unsupervised method [66] and ii) application of the singular value decomposition or least square methods. The network output is defined as below [67]:

$$Y_{q}(Z)=\sum_{s=1}^{\varepsilon }w_{qj}\varphi_{j}(\|Z-U_{j}\|)+b_{q}$$ (7)

where *Z* is the input vector and U_*j*_ is the vector, which determines the basis function (*φ*_*j*_) center. ‖*Z*−U_*j*_‖ represents the Euclidean distance between *Z* and U_*j*_. The *w*_*qj*_ and *b*_*q*_ are the weights and biases, respectively. The following Gaussian (basis) function was used:

$$\varphi_{j}(\|Z-U_{j}\|)=e^{\left(-\frac{\left|Z-U_{j}\right|}{2\sigma^{2}}\right)}$$ (8)

where *σ* is spread of the Gaussian, that controls the smoothness of the estimating function.

The abovementioned variables that seem to potentially affect hydraulic attributes were chosen as inputs and the *K*_*fs*_ as output (target) in RBFNNs. It should be pointed out that since the mentioned variables (inputs and target) used were of different orders of magnitude and with different units; therefore, the original inputs and output parameters (V_i_) were normalized (V) between 0.1 and 0.9 before presenting into the RBFNNs as follows:

$$V=[\left(V_{i}‐V_{\min})\times 0.8/(V_{\max}‐V_{\min}\right)]+0.1$$ (9)

where V_min_ and V_max_ are the minimum and the maximum values of the measured attributes (V), respectively. Luk et al. [68] also showed that the normalization of data for NNs training resulted in faster convergence and better performance.

Similar to MLR, two training and test data sets were used. The former data set was employed to determine the most appropriate *w* and *b* parameters and the latter to test the performance of NN [3,69]. The network parameters were determined according to the difference (error) between the measured and RBFNNs-predicted *K*_*fs*_ until the desired error was met [70]. In the present study, all NNs computations were performed using the MATLAB software packages (Mathworks, Natick, Massachusetts, USA, 2015).

**Fig 4:**
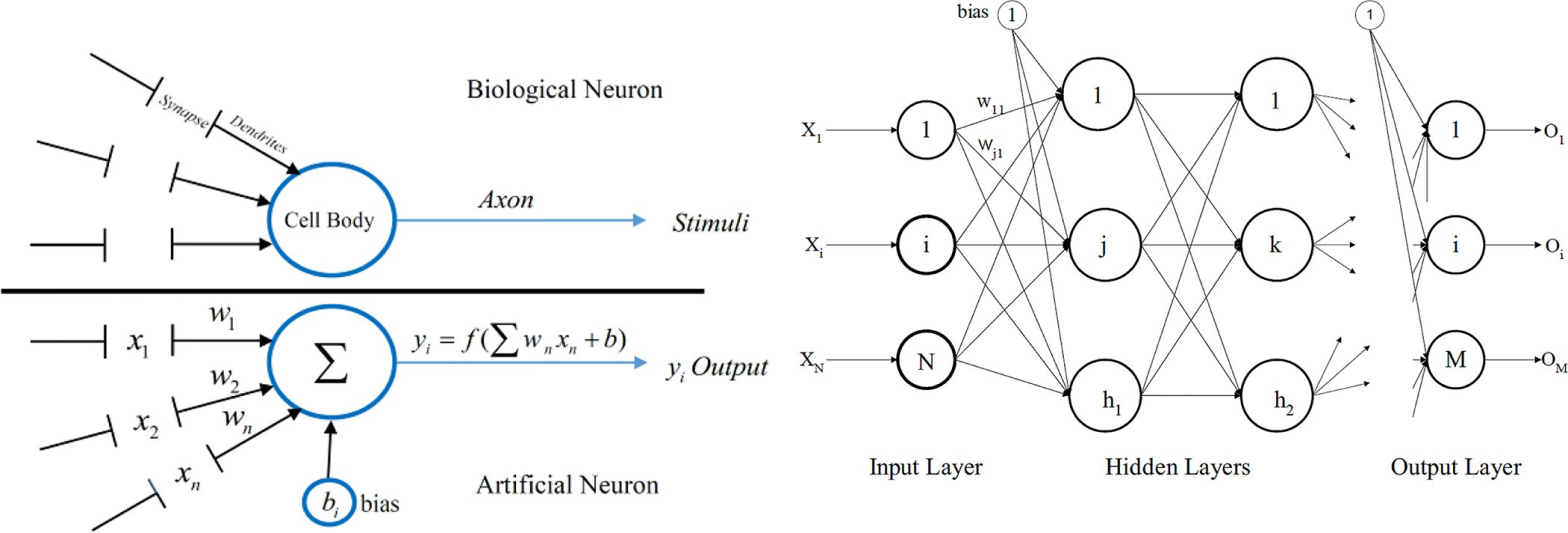
Schematic comparison between a biological neuron and an artificial neuron (a) (after López et al. [72]) along with a feed-forward network structure (b). X = input data; *w* = weights; *b* = bias value; f = transfer function.

*Multilayer perceptron neural networks (MLPNNs)*. The MLPNNs as a FFNNs are commonly applied in science and engineering applications (Fig 4). The neurons of neighboring layers are connected with *w* and *b* parameters [11,18]. The learning algorithm aims to determine the *w* and *b* parameters. In this paper, MLPNNs was applied to estimate the *K*_*fs*_ of the studied calcareous soils.

The MLPNNs are trained using an iterative back-propagation (BP) learning procedure to justify the *w* parameters as below [67,71]:

$$w_{ji}(k+1)=w_{ji}(k)+\Delta w_{ji}(k)$$ (10)

The generalized-delta-learning rule is used to compute the Δw_ji_(k). For example, for a single hidden layer network, the Δw_ji_ is determined as below:

$$\Delta w_{ji}(q)=\gamma f_{j}^{\prime }(.)x_{i}\sum_{k=1}^{K}\left\{\left[{\left(K_{fs-m}\right)}_{k}-{\left(K_{fs-p}\right)}_{k}]f_{k}^{\prime }(.)w_{kj}(q\right)\right\}+\alpha \Delta w_{ji}(q-1)$$ (11)

where *γ*, *f*′(.), *x*_*i*_, and *α* are the rate of learning, the transfer function derivative with respect to its input, the i^th^ input to NNs, and the momentum value that is a positive number between 0 and 1, respectively. *q* shows the iteration number. Maren et al. [12] and Rojas [18] give further information.

In the present study, for training MLPNNs with one and two hidden-layers including different neurons were examined. After some trials, MLPNNs with two hidden layers and 15 and 21 neurons were selected in the 1^st^ and the 2^nd^ hidden layers, respectively.

In the mentioned ANNs, the sigmoid and linear functions were employed in the hidden and output layers, respectively. The training of MLPNNs was performed with different input-output patterns until the desired error (residual) between the measured (original) and predicted *K*_*fs*_ was achieved [3,70]. Different transfer functions (e.g., log sigmoid, tangent sigmoid, etc) were applied in the hidden layers. Furthermore, different optimization (learning) algorithms (e.g., Steepest Descent Gradient, Conjugate Gradient, Levenberg–Marquardt, etc) were used.

**Fig 5:**
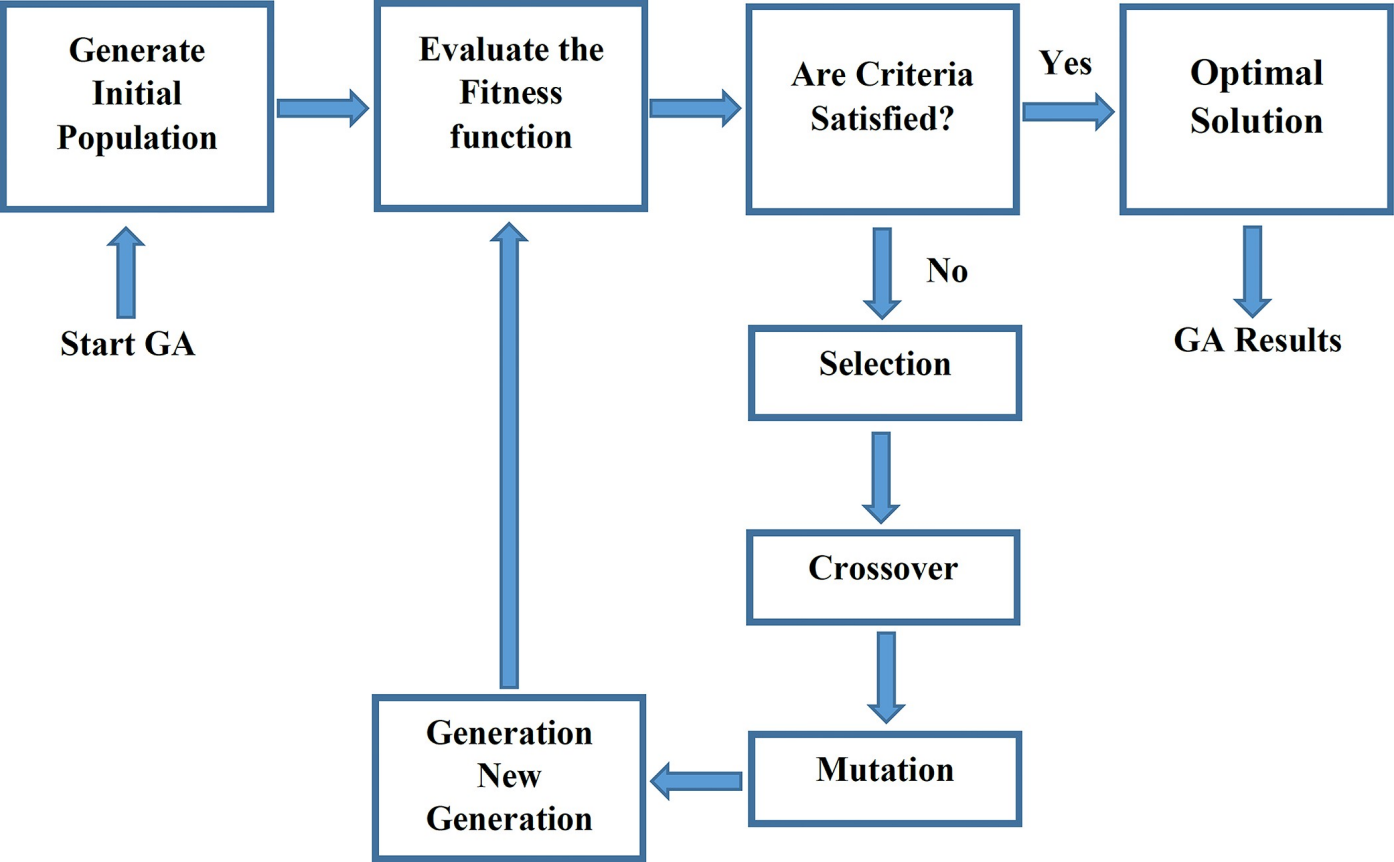
The structure of genetic algorithm (GA).

*Hybrid genetic algorithm—NNs (GA-NNs)*. The GA that is an evolutionary optimization method inspired by the nature and the process of natural selection starts with a random initial population. Then a series of operations such as selection, crossover, and mutation are performed to generate the new population and gradually evolve until the optimal solution is reached by calculating the fitness function (Fig 5). In the present study, the fitness function, which is to be minimized, defined as the mean square error (Eq 12) between the measured and NNs-predicted *K*_*fs*_ [25,45].

$$E=\frac{1}{2}\sum_{i=1}^{n}[{\left(K_{fs}\right)}_{p_{i}}-{\left(K_{fs}\right)}_{m_{i}}){]}^{2}$$ (12)

where ${{(K}_{\mathrm{fs}})}_{p_{i}}$ and ${{(K}_{\mathrm{fs}})}_{m_{i}}$ are the predicted and the measured *K*_*fs*_, respectively and *n* is the number of data presented to the NNs.

Furthermore, in this research, the maximum number of GA iterations, size of population, crossover rate, and mutation rate were selected as 600, 50, 0.9, and 0.01, respectively. In addition, the roulette wheel method was used for selection of each individual based on its fitness in the population.

**Fig 6:**
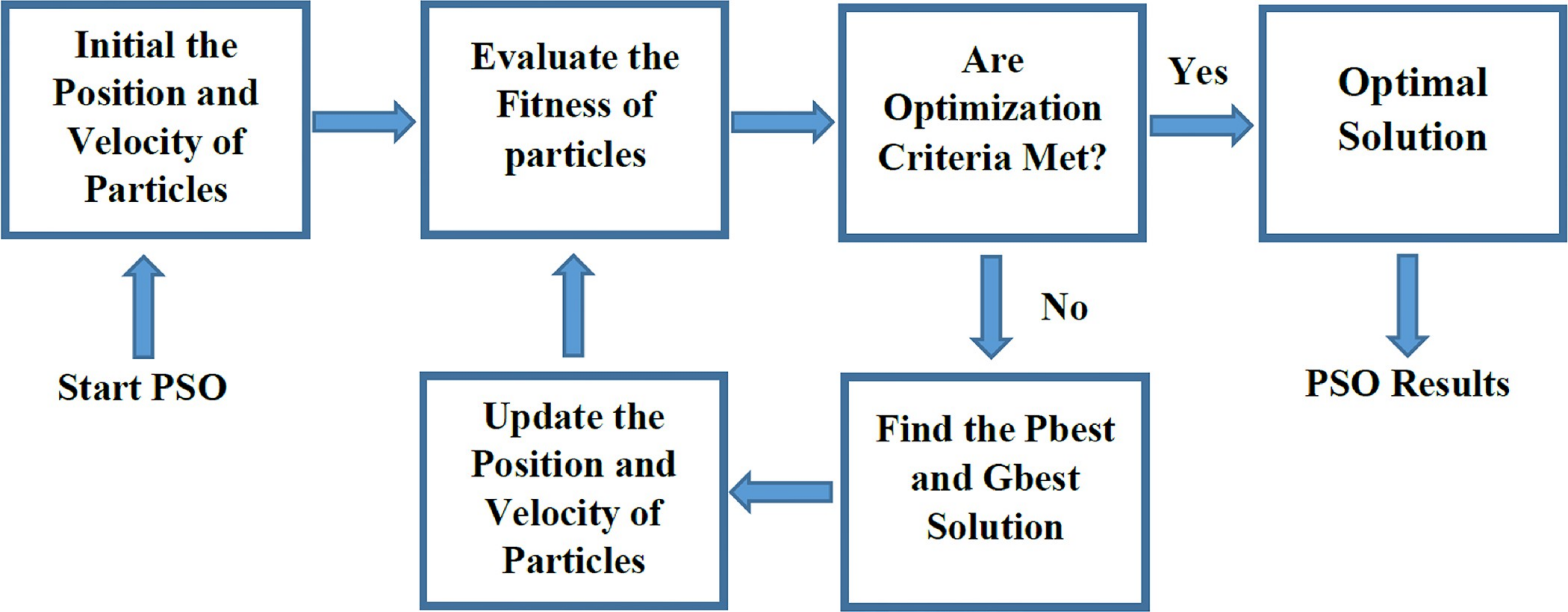
The structure of particle swarm optimization (PSO).

*Hybrid particle swarm optimization- NNs (PSO-NNs)*. The PSO algorithm is a population-inspired algorithm for solving optimization problems [27]. This algorithm is inspired by communication of fish schooling or bird flocking. The PSO has attracted much interest from many researchers due to fast convergence and simple implementation [7,3]. The PSO applies a number of particles moving around the *M* dimensional space to find the appropriate solution. Each particle *i* has a position vector $x_{i}^{t}$ and a velocity vector $v_{i}^{t}$ at time *t*. The personal best solution $P_{best_{i}^{t}}$ is achieved by an *i*^th^ particle at time *t*. The $g_{best_{i}^{t}}$ is the global best solution which is found among the personal best solutions in the whole swarm. The velocity and position of *i*^th^ particle updated at time *t*+1 as follows:

$$\begin{array}{l} v_{i}^{t+1}=\omega \times v_{i}^{t}+c_{1}\times r_{1}\times (P_{best,i}^{t}-x_{i}^{t})+c_{2}\times r_{2}\times (g_{best,i}^{t}-x_{i}^{t})\\ x_{i}^{t+1}=x_{i}^{t}+v_{i}^{t+1} \end{array}$$ (13)

where *ω*, *c*_*1*_, and *c*_*2*_ are the inertia, social, and cognitive parameters, respectively. The *r*_1_ and *r*_2_ are two uniformly distributed random numbers in (0, 1). According to Eq (13), the optimal solution is achieved by the contribution of the current velocity, the personal best solution (experience of each particle), and the global best solution (experience of other particles). Fig 6 shows the structure of PSO. In its structure, *c*_*1*_ and *c*_*2*_ are considered 1.479. The inertia weight (ω) was computed as below [74]:

$${\omega =\omega }_{\max}‐\frac{k(\omega_{\max}{‐\omega }_{\min})}{{\mathrm{Max}}_{\mathrm{iteration}}}$$ (14)

where *ω*_*max*_ and *ω*_*min*_ were considered as 0.9 and 0.4, respectively and *k* is iteration number.

In the present study to find the best weights and biases, an optimization problem was developed that minimized the difference between the NNs predicted and the measured values (Eq 12). In these algorithms (GA and PSO), different sets of *w* and *b* parameters are obtained. Then the fitness of each set of *w* and *b* parameters is evaluated to analyze the accuracy of the NNs predictions.

### Cross validation

Also, to improve the performance of all developed models (MLR, RBFNNs, MLPNNs, GA-NNs, and PSO-NNs), the k-fold (k equal to 5) cross validation method was applied. In the mentioned common approach, the entire data was divided into k partitions, randomly. Among whole partitions, one is considered as the test data and the training procedure performed by the remaining k-1 partitions. The procedure was repeated k times. In other words, each k-1 fold chosen as the data set for the training stage. Lastly, the mean error resulted from whole k folds computed [75,76]

### Performance evaluation of the models using statistical measures

In the present study, different combinations of the input variables were used to predict *K*_*fs*_. Therefore, to make the predictions comparable and choose the best inputs, some key statistical measures for evaluating the predictability performance including the mean absolute percentage error (MAPE), the root mean square error (RMSE), and the correlation coefficient (R) between the measured ($K_{fs_{m_{i}}}$) and the predicted ($K_{fs_{p_{i}}}$) *K*_*fs*_ were calculated using the following Eqs.:

$$\mathrm{MAPE}=\sum_{i=1}^{N}\frac{1}{N}\left|\frac{{{(K}_{fs})}_{p_{i}}{‐(K}_{fs}{)}_{m_{i}}}{{{(K}_{fs})}_{m_{i}})}\right|\times 100$$ (15)

$$\mathrm{RMSE}=\sqrt{\frac{1}{N}\sum_{i=1}^{N}{\left({{(K}_{fs})}_{p_{i}}{‐(K}_{fs}{)}_{m_{i}}\right)}^{2}}$$ (16)

$$R=\frac{\sum_{i=1}^{N}\left({\left(K_{fs}\right)}_{p_{i}}‐{\left({\bar{K}}_{fs}\right)}_{p})({\left(K_{fs}\right)}_{m_{i}}‐{\left({\bar{K}}_{fs}\right)}_{m}\right)}{\sqrt{\sum_{i=1}^{N}{\left({\left(K_{fs}\right)}_{p_{i}}‐{\left({\bar{K}}_{fs}\right)}_{p}\right)}^{2}\sum_{i=1}^{N}{\left(\left({\left(K_{fs}\right)}_{m_{i}}‐{\left({\bar{K}}_{fs}\right)}_{m}\right)\right)}^{2}}}$$ (17)

where ${\left({\bar{K}}_{fs}\right)}_{p}$ and ${\left({\bar{K}}_{fs}\right)}_{m}$ are the average values of the predicted and the measured *K*_*fs*_ values, respectively.

Note: This study was conducted in the Agricultural Research Station, College of Agriculture, Shiraz University, Shiraz, Iran. It should be noted that the corresponding authors are academic staff of the college and no permission was required to access the field site.

## Results and discussion

### Summary statistics of the input and output variables

Table 2 shows the summary statistics of the data. The studied soils are on average with medium textures and relatively wide ranges of physico-chemical attributes. For example, the ranges of BD, W_i_, W_s_, MWD, GMD, OM, pH, EC, CCE, SAR, clay, silt, and sand contents are 1.15–1.89 g cm^-3^, 0.55–1.69 g g^-1^, 18.72–46.81 g g^-1^, 0.50–1.04 mm, 0.52–0.85 mm, 1.09–4.42%, 7.31–7.81, 0.13–1.19 dS m^-1^, 34.38–48.50%, and 0.02–0.43 meq^0.5^ L^−0.5^, 11.88–29.88, 43.84–59.60, and 14.84–31.56% for entire data set, respectively. *K*_*fs*_ were generally very high, ranging from 1.16–6.16 cm h^-1^, 1.16–6.16 cm h^-1^, and 1.25–5.01 cm h^-1^ with CV values of 0.37, 0.36, and 0.41 for the entire, training, and test data, respectively. High values of *K*_*fs*_ in the studied area may correspond to the good structure, and high amounts of OM, CCE, and macropores. Rezaei Arshad et al. [77] also reported high values of 6.4–207 cm day^−1^ for their studied soils. They concluded that the high K values might correspond to the large area of their region, existence of macropores and good structure.

Comparison of three main groups of physical (BD, W_i_, W_s_, clay, fine silt, silt, sand, MWD, and GMD), chemical (OM, pH, CCE, EC, and SAR), and hydraulic (*K*_*fs*_) soil properties in terms of the average CV values also is summarized in Table 2 for all data sets. It is worth mentioning that, in comparing the studied soil attributes as the introduced three groups, only the statistical measure of CV that does not depend on the units of soil attributes was employed. Results indicated that in all entire, training, and test data sets, CV values were the lowest for the physical and the highest for soil hydraulic characteristics. The statistical moment was moderate for soil chemical attributes. The observations revealed that in the studied soils, *K*_*fs*_ and the physical soil attributes are the most and the least variables, respectively. The obtained results were in accordance to those of Moosavi and Sepaskhah [6] who reported that the CV values of soil hydraulic characteristics were the highest among all their studied attributes. Whereas, opposite to our results they found that chemical soil properties were the least variable attributes (the lowest CV value) properties.

In the present study, training and test (validation) data sets had relatively similar ranges (Table 2). As earlier mentioned, the soils are typically derived from alluvial parent materials that might result in such a relatively wide range in physicochemical properties. The maximum CV values for MWD, GMD, BD, EC, SAR, OM, clay, and *K*_*fs*_ were observed for the training data, whereas the highest CV values for the remaining soil attributes were observed for the test data (Table 2). In general, among the measured soil properties, SAR showed the maximum variability (with the maximum CV values of 0.61, 0.62, and 0.61 for the entire, training, and test data sets, respectively); whereas, pH showed the minimum variability (with the minimum CV values of 0.02, 0.01, and 0.02 for the entire, training, and test data sets, respectively) (Table 2). Based on the variability classes introduced by Wilding and Dress [78], for all three data sets (i.e., entire, training, and test data sets) both physical and chemical input parameters, indicated moderate (CV of 16% to 35%) variability (Table 2). Furthermore, as Table 1 indicates, the target parameter (*K*_*fs*_), with CV values of 0.33, 0.35, and 0.31 for the entire, training, and test data sets, respectively; fall in the Wilding’s moderate variability class. Similar to our data, Moosavi and Sepaskhah [6] stated that the studied soil chemical and physical attributes showed the low to moderate variability classes; whereas, the hydraulic characteristics including the *K*_*fs*_ parameter fall in the moderate to high variability classes. The results also were in accordance with the findings of Beven et al. [79,80]. Results also indicated that among physical input properties, the maximum CV values of 27%, 26%, and 31% corresponded to fine silt for the entire, training, and test data sets, respectively. The maximum CV values of 61%, 62%, and 61% corresponded to the SAR of the mentioned data sets among chemical soil properties, respectively (Table 2). This means that the mentioned soil attributes have the maximum variation among the studied soil properties in the study area.

### Predicting by multiple linear regression (MLR)

In the present study, different combinations of input variables (Table 3) including BD, W_i_, W_s_, MWD, GMD, pH, EC, CCE, SAR, and OM were used to predict *K*_*fs*_. Using different combination of independent soil attributes resulted in different prediction accuracy in terms of the applied statistical indicators. In other words, the regression equation was highly sensitive to the soil attributes used as input variables (i.e., independent soil attributes). For example, the minimum R values between the measured and MLR-predicted *K*_*fs*_ data were obtained as 0.21, 0.23, and 0.17 for the entire, training, and test data sets, respectively when fine silt, clay, and BD were used as input parameters. This reveals a poor correlation between the measured and MLR-predicted *K*_*fs*_ value in the aforementioned conditions.

**Table 3:** Statistical indices between the measured field-saturated hydraulic conductivity ($K_{fs_{m}}$) and multiple linear regression-predicted field-saturated hydraulic conductivity ($K_{fs_{p}}$) of different data sets using different input groups.

Input parameters^a^	Data sets										
	Entire data (N = 100)				Training data (N = 75)				Test data (N = 25)		
	RMSE	MAPE	R		RMSE	MAPE	R		RMSE	MAPE	R
Silt, Clay, BD, W_i_	0.90	29.02	0.37		0.95	29.78	0.31		0.99	30.59	0.28
Fine Silt, Clay, BD	0.95	31.74	0.18		0.98	31.17	0.21		0.99	33.54	0.13
Clay, BD, W_i_, pH, EC, CCE	0.79	24.68	0.63		0.78	24.15	0.64		0.81	25.19	0.63
Clay, BD, pH, EC, CCE	0.75	25.82	0.63		0.78	25.07	0.62		0.80	26.01	0.62
Clay, W_i_, pH, EC, CCE	0.80	26.96	0.55		0.83	26.50	0.56		0.73	28.40	0.38
Clay, W_i_, pH, EC, CCE, SAR	0.83	25.41	0.51		0.83	24.56	0.56		0.84	28.14	0.13
Clay, GMD, pH, EC, CCE	0.75	24.31	0.63		0.75	23.74	0.66		0.77	26.12	0.39
Clay, pH, EC, CCE	0.80	26.97	0.55		0.83	26.61	0.56		0.87	28.12	0.42
BD, W_i_, MWD	0.87	27.27	0.43		0.89	26.30	0.45		0.89	29.53	0.30
BD, W_i_, MWD, GMD	0.81	24.68	0.55		0.83	23.96	0.56		0.87	26.97	0.45
**BD, W**_**i**_ **, MWD, GMD, pH, EC, CCE**	**0.66**	**21.94**	**0.72**		**0.68**	**21.75**	**0.73**		**0.68**	**22.54**	**0.68**
W_i_, pH, EC, CCE	0.82	27.27	0.52		0.84	26.78	0.54		0.75	28.86	0.34

^a^ BD, W_i_, W_s_, MWD, GMD, pH, EC, CCE, SAR, OM, and *K*_*fs*_ is bulk density (g cm^-3^), initial water content (g g^-1^), saturated water content (g g^-1^), mean weight diameter (mm), geometric mean diameter (mm), pH of saturated paste, electrical conductivity (dS m^-1^), calcium carbonate equivalent (%), sodium adsorption ratio ((meq L^−1^)^0.5^), organic matter content (%), and field saturated hydraulic conductivity (cm h^-1^), respectively.

The maximum RMSE, that is the standard deviation of prediction errors, was obtained as 0.94, 0.97, and 0.84 for the mentioned input parameters and the data sets, respectively (Table 3). Furthermore, the maximum MAPE values were 31.51, 30.93, and 33.37 when the mentioned input parameters were used for the entire, training, and test data sets, respectively (Table 3). In other words, results revealed that the mentioned input parameters were the least suitable input variables in predicting *K*_*fs*_ parameter. Whereas, when the mentioned inputs (fine silt, clay, and BD) changed to the combination of physical and chemical properties i.e., BD, W_i_, MWD, GMD, pH, EC, and CCE (Eq 18), the performance of regression predictor for predicting the *K*_*fs*_ parameter of all data sets significantly improved. For instance, the R values between the measured and MLR-predicted *K*_*fs*_ for the entire, training, and test data sets increased to nearly 0.72, 0.73, and 0.68, respectively. Furthermore, entering the mentioned input variables resulted in the least RMSE values of 0.66, 0.68, and 0.68 and the least MAPE values of 21.94, 21.75, and 22.54 for predicting the entire, training and test data sets, respectively.

$$K_{fs}=7.29-3.15BD-1.77Wi-1.68MWD+8.97GMD-0.52pH-1.85EC+0.04CCE\;R^{2}=0.68$$ (18)

where BD, W_i_, W_s_, MWD, GMD, pH, EC, CCE, and *K*_*fs*_ are in (g cm^-3^), (g g^-1^), (g g^-1^), (mm), (mm), (-), (dS m^-1^), (%), and (cm h^-1^), respectively.

As we expected, the results revealed that by increasing the inputs from three to seven, the accuracy of MLR estimations was remarkably increased. The results were similar to the findings of Elbisy [81] who reported that the RMSE values of regression-predicted K_s_ at field conditions varied from 0.41 to 0.54. He also stated that the correlation coefficient between the measured and regression-predicted K_s_ values varied between 0.64 and 0.92. The RMSE value of 1.18 has been obtained by Schaap and Leij [82] in the prediction of K_s_ using clay, silt, sand, and BD as input variables. Furthermore, the RMSE of 1.24 in the prediction of K(h) using some basic soil attributes has been reported by Zhuang et al. [8]. Vereecken [83] also stated that the number and type of measurement methods should be considered in evaluating the methods used for prediction of K(h).

### Predicting by artificial neural network (NNs) models

For evaluating efficiency of the models applied in the present study, different statistical measures including RMSE, R, and MAPE were determined. The RMSE is the prediction error (standard deviation of residuals) and is one of the important indices of the predictability performance. RMSE is a positive value, with high and low values showing poor and good performances, respectively [3]. The MAPE shows the bias of predictions. The positive and negative MAPE values indicate overestimation and underestimation, respectively. The zero values of MAPE indicate that the predicted variables are equal to that of the measured ones. Results showed that RMSE values between the measured and RBFNNs-predicted *K*_*fs*_ varied from 0.68 to 0.99 cm h^-1^; MAPE from 22.54 to 33.54; and R from 0.13 to 0.68 by entering different input variables (Table 4). In other words, similar to those obtained for MLR, by improving the input variables from the purely physical inputs (W_i_ + BD + MWD) to combination of physical and chemical properties (BD + W_i_ + MWD + GMD + pH + EC + CCE), the performance of predictor for prediction of entire data set significantly improved (i.e., RMSE values decreased from the 0.99 to 0.68 cm h^-1^; MAPE from 33.54 to 22.54; and R increase from 0.13 to 0.68). In general, results indicated when only the W_i_, compaction (BD), and particle size distribution (MWD) indices were considered, the applied RBFNNs could not predict the *K*_*fs*_ parameter, accurately. Whereas, involving the chemical soil attributes of pH, EC, and CCE remarkably improved the model’s ability to estimate *K*_*fs*_. Moosavi and Sepaskhah [5] also stated that using saline irrigation waters with low EC values (less than 10 dS m^-1^) could improve the hydraulic characteristics of the soils relating to soil ability for water movement. They concluded that at near-saturated conditions (i.e., at applied suctions of 0 to 10 cm) the maximum values of hydraulic conductivity were obtained when water with EC of 10 dS m^-1^ was applied. Omidifard and Moosavi [84] also reported that there was a positive correlation between CCE and *K*_*fs*_ parameter of their studied soils. They concluded that calcium carbonate resulted in the flocculation of soil particles, consequently, *K*_*fs*_ increased. Therefore, in our study, the identified combination of physico-chemical suitable input parameters was selected to predict *K*_*fs*_ using the other types of NNs approaches.

**Table 4:** Statistical indices between the measured field-saturated hydraulic conductivity ($K_{fs_{m}}$) and RBFNNs-predicted field-saturated hydraulic conductivity ($K_{fs_{p}}$) of different data sets using different input groups.

	Data sets										
	Entire data (N = 100)				Training data (N = 75)				Test data (N = 25)		
Input parameters ^a^	RMSE	MAPE	R		RMSE	MAPE	R		RMSE	MAPE	R
Silt, Clay, BD, W_i_	0.61	17.11	0.78		0.46	12.24	0.91		0.93	32.71	0.01
Fine Silt, Clay, BD	0.73	21.61	0.67		1.06	15.73	0.85		0.59	40.41	0.26
Clay, BD, W_i_, pH, EC, CCE	0.25	4.20	0.97		0.08	1.07	0.99		0.49	14.22	0.80
Clay, BD, pH, EC, CCE	0.30	5.88	0.95		0.13	2.66	0.99		0.57	16.09	0.76
Clay, W_i_, pH, EC, CCE	0.26	4.61	0.96		0.10	1.60	0.99		0.51	14.23	0.77
Clay, W_i_, pH, EC, CCE, SAR	0.21	3.26	0.98		0.06	0.82	0.99		0.43	11.06	0.85
Clay, GMD, pH, EC, CCE	0.39	6.12	0.92		0.11	2.57	0.99		0.78	17.47	0.53
Clay, pH, EC, CCE	0.36	8.23	0.93		0.21	5.07	0.98		0.63	18.34	0.70
BD, W_i_, MWD	0.70	20.45	0.68		0.60	16.63	0.82		0.96	32.66	0.09
BD, W_i_, MWD, GMD	0.54	15.34	0.83		0.43	11.39	0.91		0.80	27.97	0.40
**BD, W**_**i**_ **, MWD, GMD, pH, EC, CCE**	**0.18**	**3.53**	**0.98**		**0.11**	**1.53**	**0.99**		**0.29**	**9.95**	**0.93**
W_i_, pH, EC, CCE	0.23	4.61	0.96		0.10	1.60	0.99		0.51	14.23	0.77

^a^ BD, W_i_, W_s_, MWD, GMD, pH, EC, CCE, SAR, OM, and *K*_*fs*_ is bulk density (g cm^-3^), initial water content (g g^-1^), saturated water content (g g^-1^), mean weight diameter (mm), geometric mean diameter (mm), pH of saturated paste, electrical conductivity (dS m^-1^), calcium carbonate equivalent (%), sodium adsorption ratio ((meq L^−1^)^0.5^), organic matter content (%), and field saturated hydraulic conductivity (cm h^-1^), respectively.

**Fig 7:**
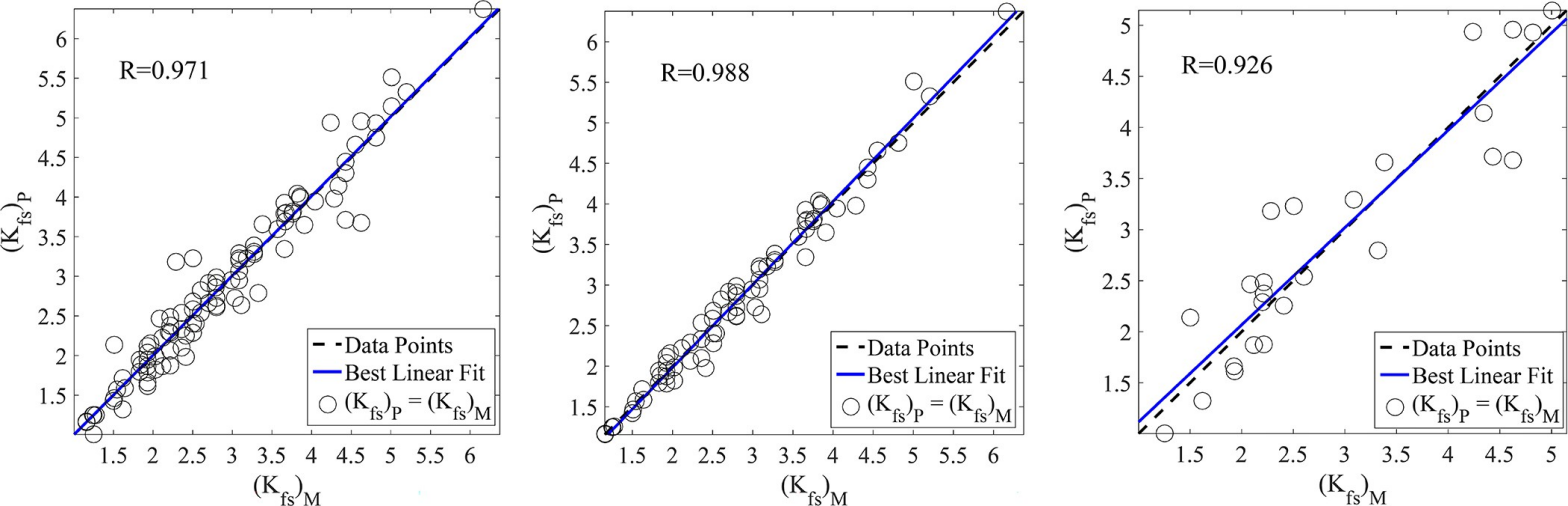
Measured vs. predicted field-saturated hydraulic conductivity, *K*_*fs*_ (cm h^-1^) data using the best RBFNNs and the most appropriate input parameters (based on those reported in Table 2) for the (a) entire, (b) training and (c) test data sets along with their correlation coefficients (R).

In the present study, the prediction of *K*_*fs*_ was assessed by the application of RBFNNs, MLPNNs, GA-NNs, and PSO-NNs. It is revealed from the results of test data set (Table 5) obtained by employing RBFNNs (R = 0.926, MAPE = 14.30, and RMSE = 0.452), MLPNNs (R = 0.933, MAPE = 12.13, and RMSE = 0.426), GA-NNs (R = 0.949, MAPE = 11.83, and RMSE = 0.404), PSO-NNs (R = 0.958, MAPE = 9.48, and RMSE = 0.343), that the PSO-NNs was found as the best model for predicting *K*_*fs*_. Figs 7 (RBFNNs), 8 (MLPNNs), 9 (GA-NNs), and 10 (PSO-NNs) also indicate that there is a highly significant correlation between the measured and NNs-predicted *K*_*fs*_ values.

**Fig 8:**
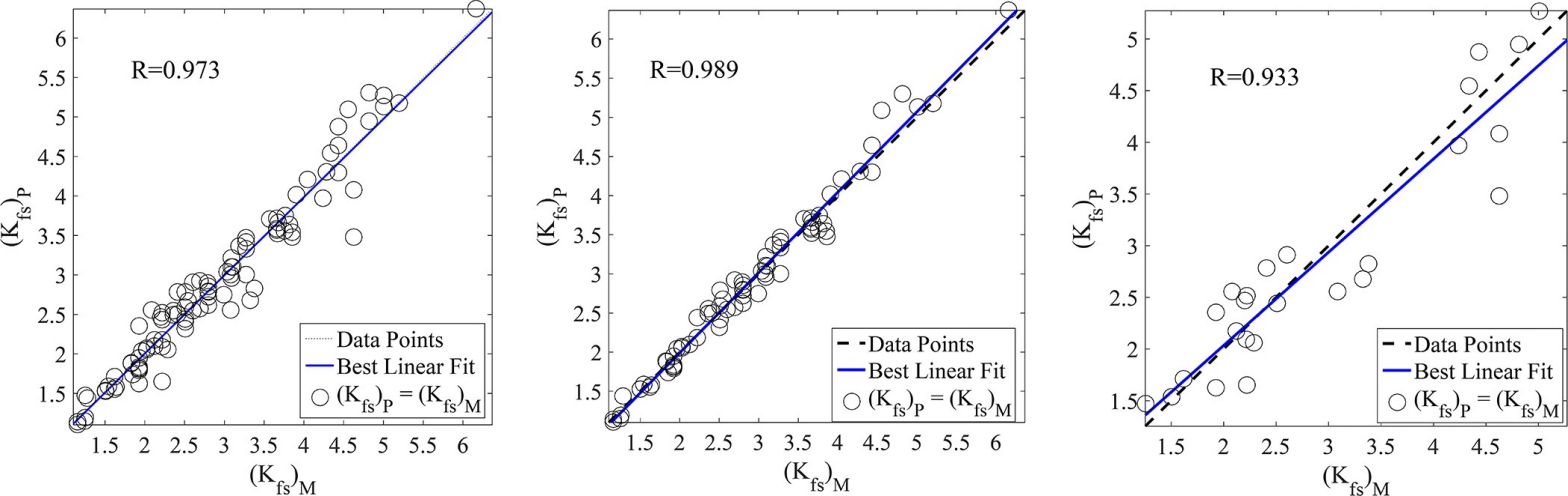
Measured vs. predicted field-saturated hydraulic conductivity, *K*_*fs*_ (cm h^-1^) data using the best MLPNNs and the most appropriate input parameters (based on those reported in Table 2) for the (a) entire, (b) training and (c) test data sets along with their correlation coefficients (R).

**Fig 9:**
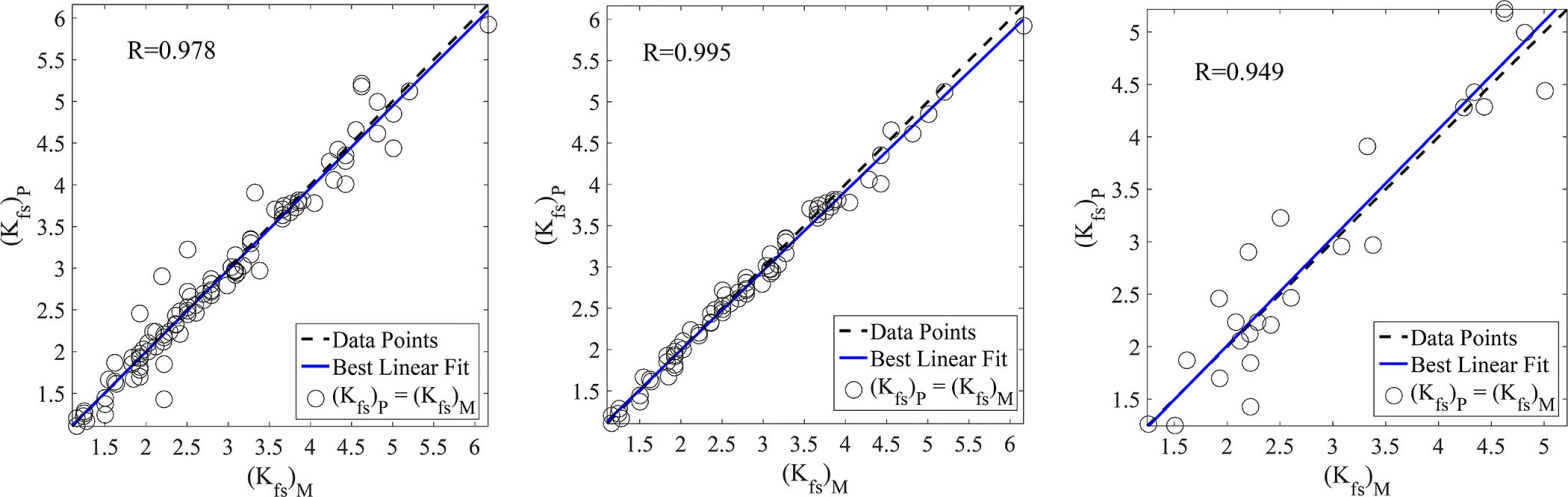
Measured vs. predicted field-saturated hydraulic conductivity, *K*_*fs*_ (cm h^-1^) data using the best GA-NNs and the most appropriate input parameters (based on those reported in Table 2) for the (a) entire, (b) training and (c) test data sets along with their correlation coefficients (R).

**Fig 10:**
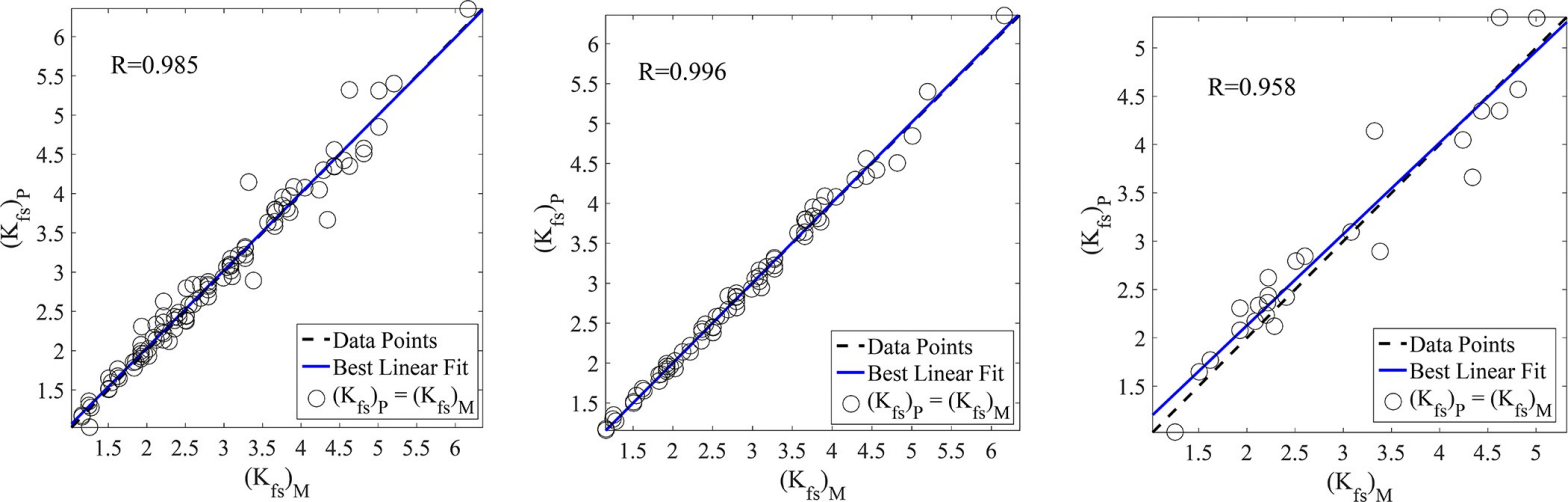
Measured vs. predicted field-saturated hydraulic conductivity, *K*_*fs*_ (cm h^-1^) data using the best PSO-NNs and the most appropriate input parameters (based on those reported in Table 2) for the (a) entire, (b) training and (c) test data sets along with their correlation coefficients (R).

**Table 5:** Comparison between the applied estimation methods in terms of statistical indices between the measured ($K_{fs_{m}}$) and predicted field-saturated hydraulic conductivity ($K_{fs_{p}}$) of different data sets using the best-proposed input group of soil variables.

	Data sets										
	Entire data set (N = 100)				Training data set (N = 75)				Testing data set (N = 25)		
Prediction methods^a^	RMSE	MAPE	R		RMSE	MAPE	R		RMSE	MAPE	R
Multiple linear regression (MLR)	0.661	21.94	0.722		0.683	21.75	0.734		0.675	22.54	0.683
Redial basis function neural networks (RBFNNs)	0.271	6.93	0.971		0.172	4.47	0.988		0.452	14.30	0.926
Multilayer perceptron neural networks (MLPNNs)	0.253	6.19	0.973		0.165	4.22	0.989		0.426	12.13	0.933
Genetic algorithm NNS (GA-NNs)	0.228	5.32	0.979		0.113	3.15	0.995		0.404	11.83	0.949
Particle swarm optimization NNs (PSO-NNs)	0.192	4.09	0.985		0.091	2.29	0.996		0.343	9.47	0.958

^a^ BD, W_i_, W_s_, MWD, GMD, pH, EC, CCE, SAR, OM, and *K*_*fs*_ is bulk density (g cm^-3^), initial water content (g g^-1^), saturated water content (g g^-1^), mean weight diameter (mm), geometric mean diameter (mm), pH of saturated paste, electrical conductivity (dS m^-1^), calcium carbonate equivalent (%), sodium adsorption ratio ((meq L^−1^)^0.5^), organic matter content (%), and field saturated hydraulic conductivity (cm h^-1^), respectively.

Although the RBFNNs are faster than the other applied models, their generalization was not good in our study. Therefore, the well-known MLPNNs are utilized to predict *K*_*fs*_. The results showed that the accuracy of MLPNNs-predictions is better than that of RBFNNs. Furthermore, the traditional learning algorithms (e.g., Levenberg-Marquardt and Conjugate Gradient) used in the MLPNNs structure may suffer from getting stuck in the local minimums. Therefore, the GA and PSO techniques were employed to justify the weights and biases in the MLPNNs. The results indicated that these approaches led to more accurate predictions (Table 5 and Figs 7–10) than that of the traditional learning algorithms.

There is no study on estimating *K*_*fs*_ parameter using NNs, therefore the results are compared to the studies used NNs for estimating the other soil properties. For instance, while predicting K_s_ Rezaei Arshad et al. [77] stated that the RBFNNs and MLPNNs had relatively similar ability in predicting saturated hydraulic conductivity. They also stated that RBFNNs were a little (*P* > 0.05) better than MLPNNs. Generally, they concluded that RBFNNs prediction was the most accurate among the employed NNs. Furthermore, Kianpoor Kalkhajeh et al. [85] reported that the application of RBFNNs for the prediction of CEC resulted in more accurate predictions than MLPNNs or traditional regression approaches. In fact, RBFNNs and MLPNNs are the most common NNs architectures for regression or classification problems. Some investigators [86] also declared that the RMSE of NNs-prediction of K in the training process was the same or less than that of the reported values in the other research. Whereas, the RMSE values of training data were nearly 0.5 times that of the Rosetta. They concluded that this might correspond the more limited range of the data as compared with the Rosetta and also due to different methods used to measure K in training data and Rosetta. Vereecken [83] also reported that the number and type of measurement methods should be considered in evaluating the methods used for prediction of K(h). Furthermore, Ines and Mohanty [87,88] accurately estimated K(h) at near-surface, 0 to 5 cm (R = 0.94) and sub-surface, 0 to 60 cm (R = 0.83) sections of their studied soils by using GA. Parasuraman et al. [47] also found that GA models with RMSE values of 0.89 to 0.90 resulted in more accurate K_s_ predictions than NNs with RMSE value of 1.04. The other investigators reported that global inverse parameter optimization approaches such as GA were powerful methods to estimate field-scale hydraulic properties of heterogeneous soils [48] and hydraulic conductivity in one- and two-dimensional cases [49]. Furthermore, some investigators developed inverse problems to predict K_s_ by using GA [52] and also by measuring the head of water by combining the finite element approach with the adaptive GA [50]. They found that the established inverse analysis approaches in combination of GA were effective tool for predicting the mentioned parameters. On the opposite, Fatehnia et al. [51] investigated that the applied GA model (R = 0.81 and RMSE = 0.341 cm s^-1^) resulted in less accurate predicted-K_s_ than the other used models (R = 0.86 and RMSE = 0.282 cm s^-1^).

To the best of our knowledge, one of the few works related to estimation of hydraulic conductivity by the PSO approach was reported by Zhang et al. [53]. They utilized the PSO approach to determine the most appropriate value of hydraulic characteristics for unsaturated soils and reported that the method outlined was appropriate for a wide range of geotechnical estimation problems. Furthermore, there are few published papers regarding the application of hybrid NNs with GA and PSO. For instance, Mishra et al. [54] applied a GA-NNs model to estimate K_s_ of the soil-bentonite mixture. They reported that the model with R-values of 0.98 and 0.97 for the training and test data sets, respectively was found to be satisfactory and agreed well with the measured values in comparison with the conventional NNs. They also concluded that their proposed model had a very good generalization capacity regarding so many various influential parameters on K_s_. Nematolahi et al. [55] also employed a fuzzy inference system (FIS) trained by both PSO and GA using some easily accessible soil attributes (e.g., OM, PSD, CCE, EC, and BD) measured at 113 sampling locations as input variables to predict K_s_. Their results revealed that the PSO-FIS model resulted in a higher efficiency (R = 0.85; RMSE = 5.2) compared to the GA-FIS (R = 0.73; RMSE = 5.5) and FIS (R = 0.39; RMSE = 8.9) models. In addition, Li et al. [56] proposed a new two-step inverse problem to predict hydraulic parameters for different soil textural classes using a hybrid vector-evaluated GA and PSO. They concluded the method was robust and excellent. Although there were some errors in their measured parameters (e.g., W_i_, W_s_, and cumulative infiltration), the model could estimate the hydraulic parameters accurately making it feasible for practical use.

### Comparison of the model’s performance

Results indicated that both RBFNNs and MLR methods resulted in more accurate *K*_*fs*_ predictions when BD, W_i_, W_s_, MWD, GMD, pH, EC, and CCE were applied as input parameters as compared with other combination of the input parameters (Tables 3 and 4). Bulk density is an index of soil compaction that directly affects *K*_*fs*._ This attribute is also recognized as one of the influential factors by Rezaei Arshad et al. [77]. Both MWD and GMD are indices of aggregate stability and size distribution. The mentioned soil attributes have been recognized as influential parameters for hydraulic conductivity by the other investigators [3,7]. Both W_i_ and W_s_ are indicators of the amount of water content in the soil that remarkably affects *K*_*fs*_. Electrical conductivity (EC), pH, and CCE are chemical soil attributes that indirectly affect *K*_*fs*_ via their influences on the formation and stability of soil structure and the related soil quality and degradation indices [84,89,90]. Singh et al. [91] also revealed that sand, clay, field capacity (FC), and wilting point (WP) moisture content of their studied soils are the most influential predictors for the prediction of saturated hydraulic conductivity using machine learning-derived pedotransfer functions (ML-PTFs).

Comparisons of the results revealed that NNs predictions were more accurate than those of the MLR (Table 5). In other words, statistical validation metrics showed that the performance of NNs is quantitatively higher than the MLR in terms of RMSE, MAPE, and R values (Table 5). So that, by using the same inputs (the mentioned suitable input variables), the R values between the measured and NNs-predicted *K*_*fs*_ parameter increased by nearly 24%, 25%, 27%, and 28% in the prediction of the test data set by RBFNNs, MLPNNs, GA-NNs, and PSO-NNs, respectively as compared to that of the MLR predictions. The R values indicated that most of the variance of the phenomenon could be captured by the proposed NNs. On the other hand, the scatter plots presented in Figs 7–10 can support the fact. Furthermore, the RMSE values of predictions decreased by 1.52, 1.61, 1.70, and 2.0 folds for the mentioned NN models, in comparison with that of the MLR, respectively. The MAPE values also decreased by 1.58, 1.86, 1.91, and 2.38 folds for predicting the test data set by the abovementioned NNs, respectively (Table 5). In general, the results showed that the proposed NNs had superiority to the conventional regression models in *K*_*fs*_ prediction. Furthermore, as results indicated among the applied NN models, the PSO-NNs with the largest R value and the smallest RMSE and MAPE values were the best model for the prediction of *K*_*fs*_. As results indicated (Table 5), the applied regression and NN models utilized for predicting *K*_*fs*_ prioritized regarding their performance as PSO-NNs > GA- NNs > MLPNNs > RBFNNs > MLR. The results are in accordance with the findings of Tamari et al. [92] and Minasny and McBratney [93] who concluded that NN models generally perform better than regression models where the number of inputs is high, mainly where the data uncertainties are small. Singh et al. [91] also predicted soil saturated hydraulic conductivity using machine learning-derived pedotransfer functions (ML-PTFs) including MLPNNs, GA, and two hybrid MLP-GA and support vector machine (SVM)-GA algorithms. They stated that among the mentioned ML-PTFs, the SVM-GA algorithm outperformed the other PTFs and the SVM-GA PTF demonstrated higher efficiency than the MLP-GA algorithm.

The traditional NNs such as MLPNNs and RBFNNs use classical optimization techniques (e.g., Levenberg-Marquardt, Steepest Descent, etc) and consequently are faster than PSO and GA. However, the classical optimization techniques may get stuck in a local minimum which is the major limitation of these algorithms. Whereas hybrid algorithms such as PSO-NNs and GA-NNs could find the global minimum, but they are slower than the traditional NNs. Furthermore, the main advantages of PSO-NNs as compared to GA-NNs are their simplicity and having fewer parameters to tune, which usually improve convergence speed [73,94].

## Conclusions

In the present study, field saturated hydraulic conductivity (*K*_*fs*_) of calcareous soils has been predicted using the MLR, RBFNNs, MLPNNs, GA-NNs, and PSO-NNs approaches. Results indicated that both RBFNNs and MLR models resulted in more accurate predictions when BD, W_i_, W_s_, MWD, GMD, pH, EC, and CCE were applied as input parameters as compared with the other combinations of the input parameters. Therefore, the mentioned parameters have been used as inputs for the other applied NN models. In general, results showed that the proposed NNs had higher performance than that of the conventional regression models. Furthermore, the PSO-NNs with the lowest RMSE and MAPE and the highest R values was recognized as the most suitable model for estimating *K*_*fs*_ among the others. The applied models are sorted as PSO-NNs > GA- NNs > MLPNNs > RBFNNs > MLR in terms of their performances (RMSE, MAPE, and R values). In general, the measures of statistical validation revealed that the performances of all NN models particularly PSO-NNs were satisfactory for all data sets. In conclusion, the capability of NNs models particularly PSO-NNs for predicting *K*_*fs*_ parameter by using limited available input soil variables as those used in this study with saving time and funds has been confirmed. Furthermore, the applied models could be used in further applications such as estimating different hydraulic, physical, chemical, or biological soil attributes. However, since the models may have different performances for other soil conditions and input variables, further evaluations may be needed to quantify the potential uncertainties and to assess the wider potential and adaptability of the suggested approaches before they are used in other geographical locations and soil conditions.

## Supporting information

S1 Data (XLS)

## References

[1] Mallants D, Mohanty BP, Vervoort A, Feyen J. Spatial analysis of saturated hydraulic conductivity in a soil with macropores. Soil Technol. 1997; 10:115–131. 10.1016/S0933-3630(96)00093-1.

[2] Reynolds WD, Elrick DE. Ponded infiltration from a single ring: I. Analysis of steady flow. Soil Sci Soc Am J. 1990; 54:1233–1241. 10.2136/sssaj1990.03615995005400050006x.

[3] Moosavi AA, Sepaskhah AR. Artificial neural networks for predicting unsaturated soil hydraulic characteristics at different applied tensions. Arch Agron Soil Sci. 2012a; 58:125–153.

[4] Saxton KE, Rawls WJ, Romberger JS, Papendick RI. Estimating generalized soil water characteristics from texture. Trans ASAE. 1986; 50:1031–1035. 10.2136/sssaj1986.03615995005000040039x.

[5] Moosavi AA, Sepaskhah AR. Determination of unsaturated soil hydraulic properties at different applied tensions and water qualities. Arch Agron Soil Sci. 2012b; 58:11–38. 10.1080/03650340.2010.503956.

[6] Moosavi AA, Sepaskhah AR. Spatial variability of physico-chemical properties and hydraulic characteristics of a gravelly calcareous soil. Arch Agron Soil Sci. 2012c; 58:631–656. 10.1080/03650340.2010.533659.

[7] Moosavi AA, Sepaskhah AR. Pedotransfer functions for prediction of near saturated hydraulic conductivity at different applied tensions in medium texture soils of a semi-arid region. Plant Know J. 2012d; 1:1–9.

[8] Zhuang J, Nakayama K, Yu GR, Miyazaki T. Predicting unsaturated hydraulic conductivity of soil based on some basic soil properties. Soil Till Res. 2001; 59:143–154. 10.1016/S0167-1987(01)00160-X.

[9] Moosavi AA, Sepaskhah AR. Sorptive number prediction of highly calcareous soils at different applied tensions using regression models. Plant Know J. 2013; 2:62–68.

[10] Minasny BJ, McBratney AB, Bristow KL. Comparison of different approaches to the development of pedotransfer functions for water retention curves. Geoderma. 1999; 93:225–253. 10.1016/S0016-7061(99)00061-0.

[11] Schaap MG, Leij FL, van Genuchten MTh. Neural network analysis for hierarchical prediction of soil hydraulic properties. Soil Sci Soc Am J. 1998; 62:847–855. 10.2136/sssaj1998.03615995006200040001x.

[12] Maren AJ, Harston CT, Pap RM. Handbook of neural computing applications. 1990; San Diego (CA): Academic Press.

[13] Granata F, Di Nunno F, Modoni G. Hybrid machine learning models for soil saturated conductivity prediction. Water, 2022a; 14(11):1729. 10.3390/w14111729.

[14] Mozaffari H, Moosavi AA, Nematollahi MA. Predicting saturated and near-saturated hydraulic conductivity using artificial neural networks and multiple linear regression in calcareous soils. PloS ONE. 2024; 19(1):e0296933. doi: 10.1371/journal.pone.0296933 38198486 PMC10781043

[15] Granata F, Di Nunno F, Najafzadeh M, Demir I. A stacked machine learning algorithm for multi-step ahead prediction of soil moisture. Hydrol. 2022b; 10(1): 10.3390/hydrology10010001.

[16] Wang X, Gao Y, Hou J, Yang J, Smits K, He H. Machine learning facilitates connections between soil thermal conductivity, soil water content, and soil matric potential. J Hydrol. 2024; 633:130950. 10.1016/j.jhydrol.2024.130950.

[17] Zakwan M, Niazkar M. A comparative analysis of data‐driven empirical and artificial intelligence models for estimating infiltration rates. Complexity, 2021; 1:9945218. 10.1155/2021/9945218.

[18] Rojas R. Neural networks: a systematic introduction. 1996; Springer-Verlag, Berlin.

[19] Park JW, Venayagamoorthy GK, Harley RG. MLP/RBF neural-networksbased online global model identification of synchronous generator. Ind Electron IEEE Trans. 2005; 52:1685e1695. 10.1109/TIE.2005.858703.

[20] Kashaninejad M, Dehghani A, Kashiri M. Modeling of wheat soaking using two artificial neural networks (MLP and RBF). J Food Eng. 2009; 91:602–607. 10.1016/j.jfoodeng.2008.10.012.

[21] Panagou EZ, Kodogiannis V, Nychas GJE. Modeling fungal growth using radial basis function neural networks: the case of the ascomycetous fungusMonascus ruber van Tieghem. Int J Food Microbiol. 2007; 117:276–286. 10.1016/j.ijfoodmicro.2007.03.010. 17521758

[22] Danandeh Mehr A, Bagheri F, Reşatoğlu R. A genetic programming approach to forecast daily electricity demand. In 13^th^ International Conference on Theory and Application of Fuzzy Systems and Soft Computing-ICAFS-2018. Springer International Publishing. 2018; 13:301–308.

[23] Kaim A, Cord AF, Volk M. A review of multi-criteria optimization techniques for agricultural land use allocation. Environ Model Soft. 2018; 105:79–93. 10.1016/j.envsoft.2018.03.031.

[24] Hajihassani M, Jahed Armaghani D, Kalatehjari R. Applications of particle swarm optimization in geotechnical engineering: a comprehensive review. Geotech Geol Eng. 2018; 36:705–722. 10.1007/s10706-017-0356-z.

[25] Johari A, Javadi AA, Habibagahi G. Modelling the mechanical behaviour of unsaturated soils using a genetic algorithm-based neural network. Comp Geotech. 2010; 38(1):2–13. 10.1016/j.compgeo.2010.08.011.

[26] Taghavifar H, Mardani A, Taghavifar L. A hybridized artificial neural network and imperialist competitive algorithm optimization approach for prediction of soil compaction in soil bin facility. Measur. 2013; 46(8):2288–2299. 10.1016/j.measurement.2013.04.077.

[27] Bui T, Nguyen T, Vo B, Nguyen T, Pedrycz W, Snásel V. Application of pParticle Swarm Optimization to create multiple-choice tests. J Inf Sci Eng. 2018; 34(6):1405–1423. 10.6688/JISE.201811_34(6).0004.

[28] Unsal E, Dane JH, Dozier GV. A genetic algorithm for predicting pore geometry based on air permeability measurement. Vadose Zone J. 2005; 4:389–397. 10.2136/vzj2004.0116.

[29] Calixto WP, Neto LM, Wu M, Kliemann HJ, de Castro SS, Yamanaka K. Calculation of soil electrical conductivity using a genetic algorithm. Comput Elec Agric. 2010; 71:1–6. 10.1016/j.compag.2009.12.002.

[30] Shin Y, Mohanty BP, Ines AV. Development of non-parametric evolutionary algorithm for predicting soil moisture dynamics. J Hydrol. 2018; 564:208–221. 10.1016/j.jhydrol.2018.07.003.

[31] Yang X, You X. Estimating parameters of van Genuchten model for soil water retention curve by intelligent algorithms. Appl Math Inf Sci. 2013; 7:1977–1983. 10.12785/amis/070537.

[32] Garg A, Garg A, Tai K, Barontini S, Stokes A. A computational intelligence-based genetic programming approach for the simulation of soil water retention curves. Transport Porous Media. 2014; 103:497–513. 10.1007/s11242-014-0313-8.

[33] Cropper W, Comerford N. Optimizing simulated fertilizer additions using a genetic algorithm with a nutrient uptake model. Ecol Model. 2005; 185:271–281. 10.1016/j.ecolmodel.2004.12.010.

[34] Luo Q, Wu J, Yang Y, Qian J, Wu J. Multi-objective optimization of long-term groundwater monitoring network design using a probabilistic Pareto genetic algorithm under uncertainty. J Hydrol. 2016; 534:352–363. 10.1016/j.jhydrol.2016.01.009.

[35] Siriwardene NR, Perera BJ. Selection of genetic algorithm operators for urban drainage model parameter optimisation. Math Comp Model. 2006; 44:415–429. 10.1016/j.mcm.2006.01.002.

[36] Chlumecký M, Buchtele J, Richta K. Application of random number generators in genetic algorithms to improve rainfall-runoff modelling. J Hydrol. 2017; 553:350–355. 10.1016/j.jhydrol.2017.08.025.

[37] Seyedpour SM, Kirmizakis P, Brennan P, Doherty R, Ricken T. Optimal remediation design and simulation groundwater flow coupled to contaminant transport using genetic algorithm and radial point collocation method (RPCM). Sci Total Environ. 2019; 669:389–399. 10.1016/j.scitotenv.2019.01.409. 30884263

[38] Fontan M, Ndiaye A, Breysse D, Bos F, Fernandez C. Soil–structure interaction: Parameters identification using particle swarm optimization. Comput Struc. 2011; 89:1602–1614. 10.1016/j.compstruc.2011.05.002.

[39] Kalatehjari R, Ali N, Hajihassani M, Fard MK. The application of particle swarm optimization in slope stability analysis of homogeneous soil slopes. Inter Rev Model Simul. 2012; 5:458–465.

[40] Sadoghi J, Kalantary F, SadoghiYazdi H. Calibration of soil model parameters using particle swarm optimization. Inter J Geomech. 2012; 12:229–238. 10.1061/(ASCE)GM.1943-5622.0000142.

[41] Ye X, Chen B, Jing L, Zhang B, Liu Y. Multi-agent hybrid particle swarm optimization (MAHPSO) for wastewater treatment network planning. J Environ Manage. 2019; 234:525–536. doi: 10.1016/j.jenvman.2019.01.023 30654244

[42] Zhang YL, Gallipoli D, Augarde C. Parameter identification for elasto-plastic modelling of unsaturated soils from pressuremeter tests by parallel modified particle swarm optimization. Comput Geotech. 2013; 48:293–303. 10.1016/j.compgeo.2012.08.004.

[43] Andrab SG, Hekmat A, Yusop ZB. A review: Evolutionary computations (GA and PSO) in geotechnical engineering. Comput Water Energy Environ Eng. 2017; 6:154–74. 10.4236/cweee.2017.62012.

[44] Bahrami S, Doulati Ardejani F. Application of artificial neural network and genetic algorithm to modelling the groundwater inflow to an advancing open pit mine. J Mining Environ. 2014; 6:21–30. 10.1016/j.jhydrol.2016.03.002. 25186026

[45] Hosseini M, Naeini SA, Dehghani AA, Khaledian Y. Estimation of soil mechanical resistance parameter by using particle swarm optimization, genetic algorithm and multiple regression methods. Soil Till Res. 2016; 157:32–42. 10.1016/j.still.2015.11.004.

[46] Rastgou M, He Y, Jiang Q. Implementation and efficient evaluation of backpropagation network training algorithms in parametric simulations of soil hydraulic conductivity curve. J Hydrol. 2024; 636:131302. 10.1016/j.jhydrol.2024.131302.

[47] Parasuraman K, Elshorbagy A, Bing CS. Estimating saturated hydraulic conductivity using genetic programming. Soil Sci Soc Am J. 2007; 71:1676–1684. 10.2136/sssaj2006.0396.

[48] Schneider S, Jacques D, Mallants D. Inverse modeling with a genetic algorithm to derive hydraulic properties of a multi-layered forest soil. Soil Res. 2013; 51:372–389. 10.1071/SR13144.

[49] Ushijima TT, Yeh WW. Experimental design for estimating unknown hydraulic conductivity in an aquifer using a genetic algorithm and reduced order model. Adv Water Resour. 2015; 86:193–208. 10.1016/j.advwatres.2015.09.029.

[50] Deng X, Fang H, Chen J, Kou J. Identification of hydraulic conductivity in aquifer for coupled FEM and adaptive genetic algorithm. Math Problems Engin. 2015; ID 909465. 10.1155/2015/909465.

[51] Fatehnia M, Tawfiq K, Ye M. Estimation of saturated hydraulic conductivity from double-ring infiltrometer measurements. Europ J Soil Sci. 2016; 67:135–147. 10.1111/ejss.12322.

[52] Bartlewska-Urban M, Strzelecki T. Application of genetic algorithms for the estimation of hydraulic conductivity. Studia Geotech Mech. 2018; 40:140–146. 10.2478/sgem-2018-0013.

[53] Zhang Y, Augarde C, Gallipoli D. Identification of hydraulic parameters for unsaturated soils using particle swarm optimization. 2008; In Proc 1^st^ Europ Conf Unsat Soils. (pp. 765–771).

[54] Mishra AK, Kumar B, Dutta J. Prediction of hydraulic conductivity of soil bentonite mixture using hybrid-ANN approach. J Environ Inform. 2016; 27:98–105. 10.3808/jei.201500292.

[55] Nematolahi M, Jalali V, Mehrizi MH. Predicting saturated hydraulic conductivity using particle swarm optimization and genetic algorithm. Arab J Geosci. 2018; 16:473. 10.1007/s12517-018-3846-2.

[56] Li YB, Liu Y, Nie WB, Ma XY. Inverse modeling of soil hydraulic parameters based on a hybrid of vector-evaluated genetic algorithm and particle swarm optimization. Water. 2018; 10(1):84. 10.3390/w10010084.

[57] Abtahi A, Karimian N, Solhi M. Semi quantified soil science report of Badjah, Fars Province. Department of Soil Science, College of Agriculture, Shiraz University, Shiraz, Iran. 1992.

[58] GSI: Geological Survey and Mineral Exploration of Iran. 2017. Geological map (1:100000).

[59] Soil Survey Staff. Keys to Soil Taxonomy, 12^th^ Ed. 2014; USDA-Natural Resources Conservation Service, Washington, DC.

[60] Omidifard M. Spatial variability of near-saturated soil hydraulic conductivity, soil moisture characteristic curve and water infiltration equations parameters, and development of pedotransfer functions for their estimation. 2014; M.Sc. thesis, Department of Soil Science, Shiraz University.

[61] Gavili E, Moosavi AA, Moradi Choghamarani F. Cattle manure biochar potential for ameliorating soil physical characteristics and spinach response under drought. Arch Agron Soil Sci. 2018; 64:1714–1727. 10.1080/03650340.2018.1453925.

[62] Baghernejad M, Javaheri F, Moosavi AA. Adsorption isotherms of copper and zinc in clay minerals of calcareous soils and their effects on x-ray diffraction. Arch Agron Soil Sci. 2015; 61:1061–1077. 10.1080/03650340.2014.982549.

[63] Baghernejad M, Javaheri F, Moosavi AA. Adsorption isotherms of some heavy metals under conditions of their competitive adsorption onto highly calcareous soils of southern Iran. Arch Agron Soil Sci. 2016; 62:1462–1473. 10.1080/03650340.2016.1147647.

[64] Zahedifar M, Dehghani S, Moosavi AA, Gavili E. Temporal variation of total and DTPA-extractable heavy metal contents as influenced by sewage sludge and perlite in a calcareous soil. Arch Agron Soil Sci. 2017; 63:136–149. 10.1080/03650340.2016.1193164.

[65] Khan I, Zakwan M, Pulikkal AK, Lalthazula R. Impact of unplanned urbanization on surface water quality of the twin cities of Telangana state, India. Marine Pollut Bullet. 2022; 185: 114324. 10.1016/j.marpolbul.2022.114324.

[66] Moody JE, Darken CJ. Fast learning in networks of locally tuned processing units. Neur Comput 1989; 1:281–294. 10.1162/neco.1989.1.2.281.

[67] Nematollahi MA, Mousavi Khaneghah A. Neural network prediction of friction coefficients of rosemary leaves. J Food Process Eng. 2019; 42(6):e13211. 10.1111/jfpe.13211.

[68] Luk KC, Ball JE, Sharma A. A study of optimal model lag and spatial inputs to artificial neural network for rainfall forecasting. J Hydrol. 2000; 227:56–65. 10.1016/S0022-1694(99)00165-1.

[69] Gautam MR, Zhu J, Ye M. Regularized artificial neural network training for biased data of soil hydraulic parameters. Soil Sci. 2011; 176:1–9. 10.1097/SS.0b013e3182316c93.

[70] Balkhair KS. Aquifer parameters determination for large diameter wells using a neural network approach. J Hydrol. 2002; 265:118–128. 10.1016/S0022-1694(02)00103-8.

[71] Nematollahi MA, Jamali B, Hosseini M. Fluid velocity and mass ratio identification of piezoelectric nanotube conveying fluid using inverse analysis. Acta Mech. 2020; 231(2):683–700. 10.1007/s00707-019-02554-0.

[72] Montesinos López OA, Montesinos López A, Crossa J. Fundamentals of Artificial Neural Networks and Deep Learning. In: Multivariate Statistical Machine Learning Methods for Genomic Prediction. Springer, Cham. 2022; 10.1007/978-3-030-89010-0_10. 36103587

[73] Xu XX, Gong HL, Ding XQ. Investigation and comparison of inertia weight control schemes in particle swarm Optimization. *In 2023 15th International Conference on Advanced Computational Intelligence (ICACI)* (pp. 1–8). IEEE.

[74] Alam MN, Das B, Pant V. A comparative study of metaheuristic optimization approaches for directional overcurrent relays coordination. Electric Power Sys Res. 2015;128:39–52. 10.1016/j.epsr.2015.06.018.

[75] Huang YW, Chen MQ, Li Y, Guo J. Modeling of chemical exergy of agricultural biomass using improved general regression neural network. Energy. 2016;114:1164–1175. 10.1016/j.energy.2016.08.090.

[76] Chatterjee S, Dey N, Sen S. Soil moisture quantity prediction using optimized neural supported model for sustainable agricultural applications. Sustain Comput Inform Sys. 2020; 28:100279. 10.1016/j.suscom.2018.09.002.

[77] Rezaei Arshad R, Sayyad Gh, Mosaddeghi M, Gharabaghi B. Predicting saturated hydraulic conductivity by artificial intelligence and regression models. Hind Pub Corpor Soil Sci. 2013; 308159:1–8. 10.1155/2013/308159.

[78] Wilding LP, Dress LR. Spatial variability and pedology. In: Wilding LP, Smeckand NE, Hall GF, (Eds.), Pedogenesis and soil Taxonomy. I. concept and Intractions. Elsevier Sci. Pub. 1983:83–116.

[79] Beven KJ, Henderson DE, Reeves AD. Dispersion parameters for undisturbed partially saturated soil. J Hydrol. 1993; 143:19–43. 10.1016/0022-1694(93)90087-P.

[80] Kilic K, Ozgoz E, Akbas F. Assessment of spatial variability in penetration resistance as related to some soil physical properties of two fluvents in Turkey. Soil Till Res. 2004; 76:1–11. 10.1016/j.still.2003.08.009.

[81] Elbisy MS. Support vector machine and regression analysis to predict the field hydraulic conductivity of sandy soil. KSCE J Civil Eng. 2015; 19:2307–2316. 10.1007/s12205-015-0210-x.

[82] Schaap MG, Leij FL. Improved prediction of unsaturated hydraulic conductivity with the Mualem-van Genuchten model. Soil Sci Soc Am J. 2000; 64:843–851. 10.2136/sssaj2000.643843x.

[83] Vereecken H. Comment on the paper, ‘‘Evaluation of pedotransfer functions for unsaturated soil hydraulic conductivity using an independent data set”. Geoderma. 2002; 108:145–147. 10.1016/S0016-7061(01)00037-4.

[84] Omidifard M, Moosavi AA. Prediction of some hydraulic properties of calcareous soils of Bajgah region Fars province using regression pedotransfer functions. Soil Res. 2015; 29:83–92. (In Persian). 10.22092/ijsr.2015.101395.

[85] Kianpoor Kalkhajeh Y, Rezaie Arshad R, Amerikhah H, Sami M. Comparison of multiple linear regressions and artificial intelligence-based modeling techniques for prediction the soil cation exchange capacity of Aridisols and Entisols in a semiarid region. Aust J Agric Eng. 2012; 3:39–46.

[86] Minasny BJ, Hopmans W, Harter T, Eching SO, Tuli A, Denton MA. Neural networks prediction of soil hydraulic functions for alluvial soils using multistep outflow data. Soil Sci Soc Am J. 2004; 68:417–429. 10.2136/sssaj2004.0417.

[87] Ines AV, Mohanty BP. Near-surface soil moisture assimilation for quantifying effective soil hydraulic properties using genetic algorithm: 1. Conceptual modeling. Water Resour Res. 2008; 44(6):W06422, 10.1029/2007WR005990.

[88] Ines AV, Mohanty BP. Near-surface soil moisture assimilation for quantifying effective soil hydraulic properties using genetic algorithms: 2. Using airborne remote sensing during SGP97 and SMEX02. Water Resour Res. 2009; 45(1):W01408, 10.1029/2008WR007022.

[89] Zahedifar M. Assessing alteration of soil quality, degradation, and resistance indices under different land uses through network and factor analysis. Catena. 2023a; 222:106807–0. 10.1016/j.catena.2022.106807.

[90] Zahedifar M. Feasibility of fuzzy analytical hierarchy process (FAHP) and fuzzy TOPSIS methods to assess the most sensitive soil attributes against land use change. Environ Earth Sci. 2023b; 82:1–17. 10.1007/s12665-023-10934-y.

[91] Singh VK, Panda KC, Sagar A, Al-Ansari N, Duan HF, Paramaguru PK,… Elbeltagi A. Novel Genetic Algorithm (GA) based hybrid machine learning-pedotransfer Function (ML-PTF) for prediction of spatial pattern of saturated hydraulic conductivity. Engin Appl Comput Fluid Mech. 2022; 16(1):1082–1099. 10.1080/19942060.2022.2071994.

[92] Tamari S, Wösten JHM, Ruiz-Suarez JC. Testing an artificial neural network for predicting soil hydraulic conductivity. Soil Sci Soc Am J. 1996; 60:1732–1741. 10.2136/sssaj1996.03615995006000060018x.

[93] Minasny B, McBratney AB. Theneuro-m method for fitting neural network parametric pedotransfer functions. Soil Sci Soc Am J. 2002; 66:352–361. 10.2136/sssaj2002.0352.

[94] Zhao M, Zhao H, Zhao M. Particle swarm optimization algorithm with adaptive two-population strategy. IEEE Access. 2023; 11:62242–62260. 10.1109/ACCESS.2023.3287859.

